# Influence of beam pruning techniques on LET and RBE in proton arc therapy

**DOI:** 10.3389/fonc.2023.1155310

**Published:** 2023-09-05

**Authors:** Helge Henjum, Johannes Tjelta, Lars Fredrik Fjæra, Sara Pilskog, Camilla H. Stokkevåg, Erlend Lyngholm, Andreas H. Handeland, Kristian S. Ytre-Hauge

**Affiliations:** ^1^ Department of Physics and Technology, University of Bergen, Bergen, Norway; ^2^ Department of Oncology and Medical Physics, Haukeland University Hospital, Bergen, Norway

**Keywords:** proton arc therapy (PAT), LET, RBE, optimization, pruning

## Abstract

**Introduction:**

Proton arc therapy (PAT) is an emerging treatment modality that holds promise to improve target volume coverage and reduce linear energy transfer (LET) in organs at risk. We aimed to investigate if pruning the highest energy layers in each beam direction could increase the LET in the target and reduce LET in tissue and organs at risk (OAR) surrounding the target volume, thus reducing the relative biological effectiveness (RBE)-weighted dose and sparing healthy tissue.

**Methods:**

PAT plans for a germinoma, an ependymoma and a rhabdomyosarcoma patient were created in the Eclipse treatment planning system with a prescribed dose of 54 Gy(RBE) using a constant RBE of 1.1 (RBE_1.1_). The PAT plans was pruned for high energy spots, creating several PAT plans with different amounts of pruning while maintaining tumor coverage, denoted PX-PAT plans, where X represents the amount of pruning. All plans were recalculated in the FLUKA Monte Carlo software, and the LET, physical dose, and variable RBE-weighted dose from the phenomenological Rørvik (ROR) model and an LET weighted dose (LWD) model were evaluated.

**Results and discussion:**

For the germinoma case, all plans but the P6-PAT reduced the mean RBE-weighted dose to the surrounding healthy tissue compared to the PAT plan. The LET was increasingly higher within the PTV for each pruning iteration, where the mean LET from the P6-PAT plan was 1.5 keV/μm higher than for the PAT plan, while the P4- and P5-PAT plans provided an increase of 0.4 and 0.7 keV/μm , respectively. The other plans increased the LET by a smaller margin compared to the PAT plan. Likewise, the LET values to the healthy tissue were reduced for each degree of pruning. Similar results were found for the ependymoma and the rhabdomyosarcoma case. We demonstrated a PAT pruning technique that can increase both LET and RBE in the target volume and at the same time decreased values in healthy tissue, without affecting the target volume dose coverage.

## 1 Introduction

Proton arc therapy (PAT) is an emerging cancer treatment modality that has shown potential to both improve target volume coverage and reduce the biological effect of protons in organs at risk (OARs) (1). Although PAT produces a larger low dose bath to the healthy tissue compared to conventional intensity-modulated proton therapy (IMPT), studies have demonstrated that the integral dose can be reduced in PAT compared to IMPT (2, 3).

In clinical proton therapy, a relative biological effectiveness (RBE) of 1.1 is applied (4) – and also used in most PAT planning studies (5–9). Nevertheless, the RBE is a variable quantity that depends on multiple factors such as the linear energy transfer (LET), tissue type and fraction dose. The LET, which is the predominant factor that influences the RBE, increases rapidly as protons slow down, reaching a maximum value a few millimeters distal of the Bragg peak. This can lead to a higher RBE in healthy tissue compared to the target volume, potentially increasing the radiation damage to healthy cells. This concern is reflected by the increasing amount of studies indicating an increased probability of normal tissue damage in areas of high LET (10–21). Due to the concerns regarding the variable RBE, multiple models and LET re-distribution techniques have been developed aiming to account for the variable RBE (22–31).

Although all variable RBE models agree on an elevated RBE towards the distal end of the beam (32), the uncertainty in the models leads to a recommendation of still prescribing a constant RBE of 1.1 clinically, while still acquiring RBE information in the clinics to review potential biological effects (33). Reviewing RBE effects could be especially useful in PAT, where the increased degrees of freedom compared to IMPT gives a high number of dose manipulation possibilities without sacrificing tumor coverage.

Several studies have investigated the LET and RBE distributions in both the target volume and OARs for PAT and IMPT. Fager et al. (34) showed how an IMPT plan optimized to sub-targets could avoid high LET values in surrounding OARs. An approach using several monoenergetic arcs demonstrated that higher LET values could be achieved in the PTV compared to an IMPT plan (35) (36). Similarly, Toussaint et al. (3) also found a higher LET in the target volume in PAT compared to IMPT and lower values outside the target volume in the OARs for an increasing number of beams. Further, Li et al. (37) used additional LET objectives in PAT to increase LET in the target volume and reduce it to the healthy tissue, while still maintaining an RBE of 1.1 in the target volume.

Since PAT utilizes proton beams from multiple angles, the highest energy layers can often be pruned without affecting the target volume dose coverage. Pruning energy layers in proton beams also shifts the high LET values in the proximal direction, potentially redistributing the high LET from the normal tissue into the target volume. The purpose of this study was therefore to explore pruning techniques, and how different amounts of pruning could alter the LET and RBE in the target volume, surrounding healthy tissue and OARs.

## 2 Method and materials

### 2.1 Treatment planning

**Figure 1:**
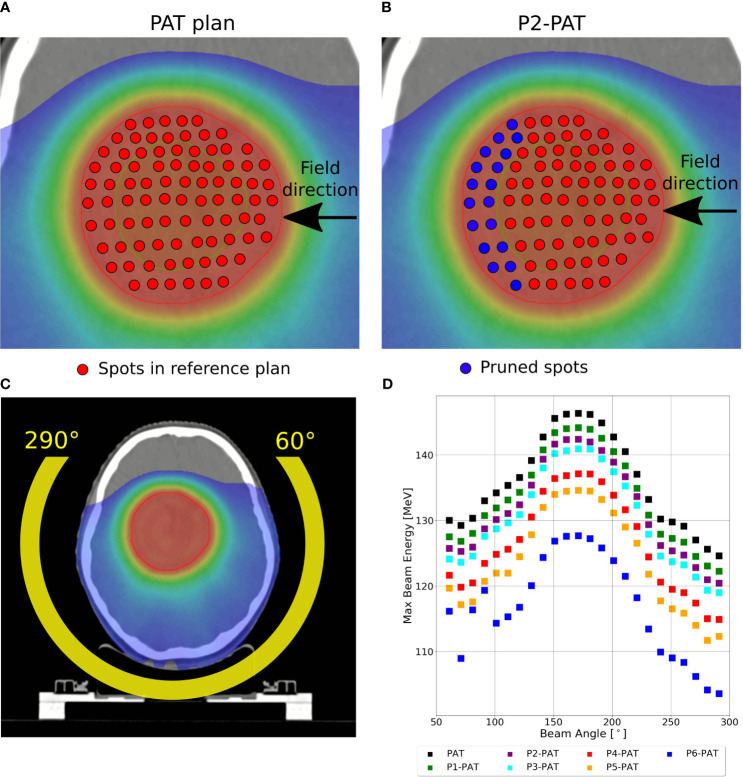
**(A)** Illustration of a spot map for a PAT plan, and **(B)** the P2-PAT plan where the blue color represents the pruned spots in this map. The red outline shows the PTV, and the green outline shows the shrunken PTV volume. The margins created for the shrunken PTV in both lateral and proximal direction makes the PTV smaller in only the distal part. **(C)** shows the beam positioning for the arc around the tumor volume and **(D)** shows the maximum energy from each beam in the arc.

To demonstrate the novel technique of PAT pruning, a germinoma case, an ependymoma case and a rhabdomyosarcoma case were investigated. The germinoma and ependymoma had a prescribed dose of 54 Gy(RBE) in 30 fractions while the rhabdomyosarcoma case had a prescribed dose of 50.4 Gy(RBE) in 28 fractions. Due to the symmetrical shape of the germinoma target volume this was chosen as a primary analysis while the ependymoma and rhabdomyosarcoma were analyzed to demonstrate the technique on more complicated target volumes. The optimization criteria in terms of PTV coverage were 95% of the prescribed dose to 100% of the target volume and 107% of the prescribed dose to 0% of the target volume ([V95%, V107%]). Different multifield optimized (MFO) plans were created in the Eclipse treatment planning system (TPS) (Varian Medical Systems, Palo Alto, California, US) according to the following criteria:

- A PAT plan with no beams positioned anterior of the patient. The separation of the beam angles varied due to difference in target volume, as the germinoma case consisted of the largest tumor volume.
  ○ Germinoma: 240 degrees arc of beams with a 10-degree separation (**Figure 1C**).
  ○ Ependymoma: 240 degrees arc of beams with a 20-degree separation.
  ○ Rhabdomyosarcoma: 260 degrees arc of beams with a 20-degree separation.
- Multiple P-PAT plans with the same setup as the PAT plan, but where high energy spots in the beams were pruned. The pruned PAT plans are denoted PX-PAT, where X represents the degree of pruning (1-6). All plans were optimized for prescribed dose after the pruning according to the optimization criteria.

The P-PAT plans were created by shrinking the original PTV in all directions equivalent to approximately one energy layer and creating margins for the new target volume in all directions except for the distal part, i.e. the shrunken PTV becomes smaller only in the distal direction for each beam. This is visualized in **Figures 1A, B**, where we see an illustration of how the spot map changes with 2 degrees of pruning. The amount of pruning for each iteration was approximately one energy layer, but varied for the individual beams as seen in **Figure 1D**.

- A reference IMPT plan was also created for all cases with the following field setups:
  ○ Germinoma: Two opposing fields.
  ○ Ependymoma: Two opposing fields, and one posterior field.
  ○ Rhabdomyosarcoma: Three oblique fields.

### 2.2 LET and variable RBE dose calculation

The plans were recalculated in our FLUKA (38, 39) MC-based recalculation system (40). We scored the dose-averaged LET (LET_d_), as described by Fjæra et al. (40). All plans were normalized to match the median dose in the PTV to the prescribed dose. To visualize biological effects, the plans were recalculated using three different RBE models: a constant RBE of 1.1 (for reference), the Rørvik (ROR) model (31), and an LET-weighted (LWD) model ( $LWD=1+cLET_{d}$ ). The ${\left(\alpha /\beta \right)}_{x}$ used in the germinoma and ependyoma case were 10 Gy and 2 Gy for the PTV and normal tissue, respectively (41, 42), while values of 2.6 Gy and 2 Gy were used for the PTV and the normal tissue in the rhabdomyosarcoma case, respectively (43). The normalization factor ( c ) for the LWD model was 0.055 μm/keV which is a value suggested by McMahon to reduce RBE variability (44). More information about the models can be found in our previous study (45).

We looked at OARs in close proximity to the target volume, including the left hippocampus and the right optic nerve. Additionally, to evaluate the overall effect of LET and RBE-weighted dose in regions surrounding the target volume, a 2 cm thick spherical shell structure was created in Eclipse, completely surrounding the PTV and is denoted further as surrounding healthy tissue. We focused on LET_d_ values, target volume coverage and maximum and mean RBE-weighted dose to the surrounding healthy tissue and OARs. The high dose and LET_d_ region in this study were considered through the dose and LET_d_ metrics for 10% and 2% of the volume. Considering that the risk of radiation induced normal tissue damage depends both on the LET_d_ and physical dose (13), different cutoff values for the LET_d_, where we only look at LET_d_ values in volumes receiving dose above a certain limit (2, 10, 30 and 50 Gy(RBE)), were also introduced to highlight regions that received simultaneously high dose and LET_d_.

## 3 Results

### 3.1 Germinoma

**Figure 2:**
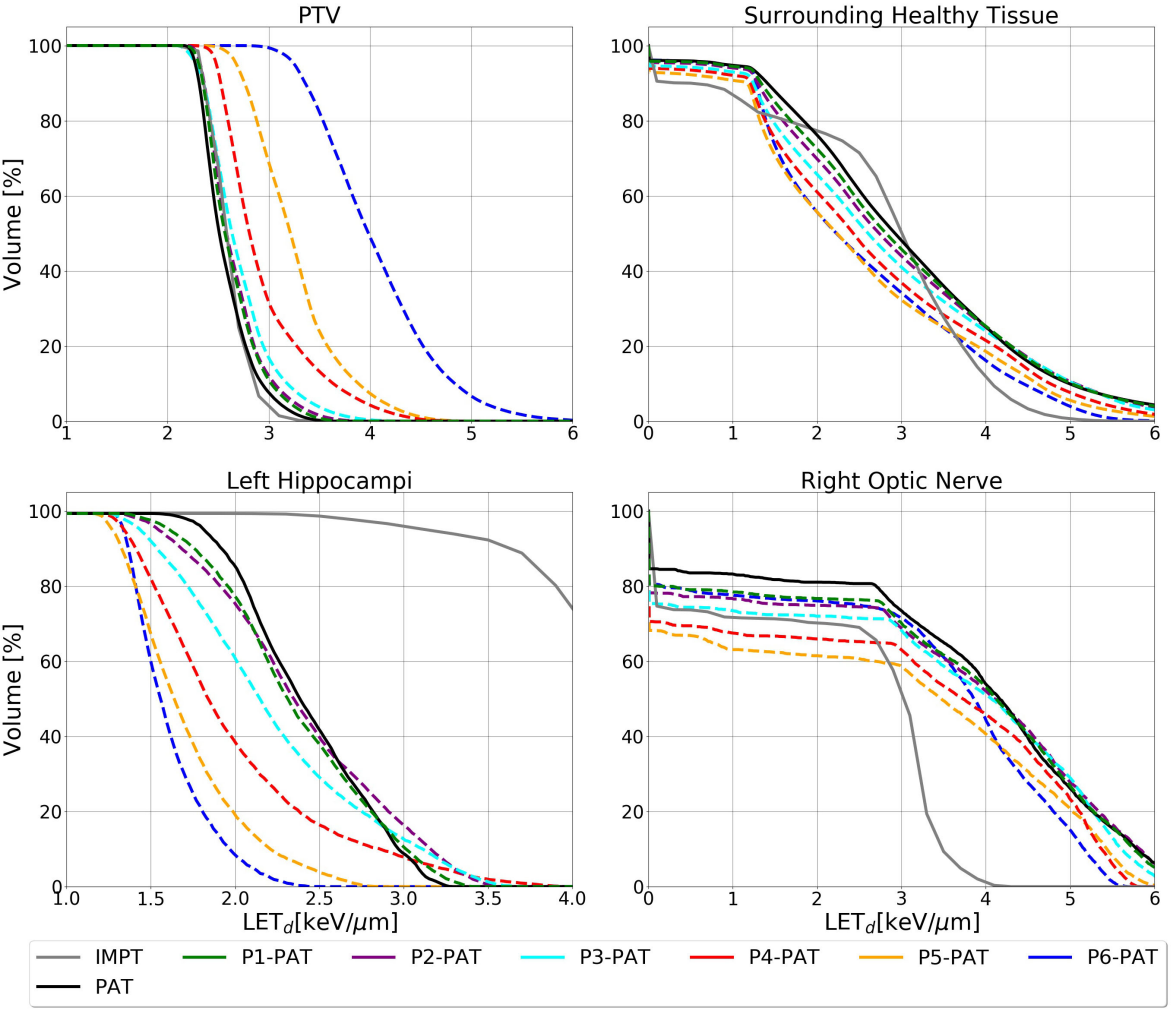
LET_d_ volume histogram for the PTV, surrounding healthy tissue and the OARs calculated with 2 Gy(RBE_1.1_) dose cutoffs for the germinoma case. The dashed lines represent the P-PAT plans, while the solid black lines represent the PAT plan and the solid gray line represent the IMPT plan.

**Figure 3:**
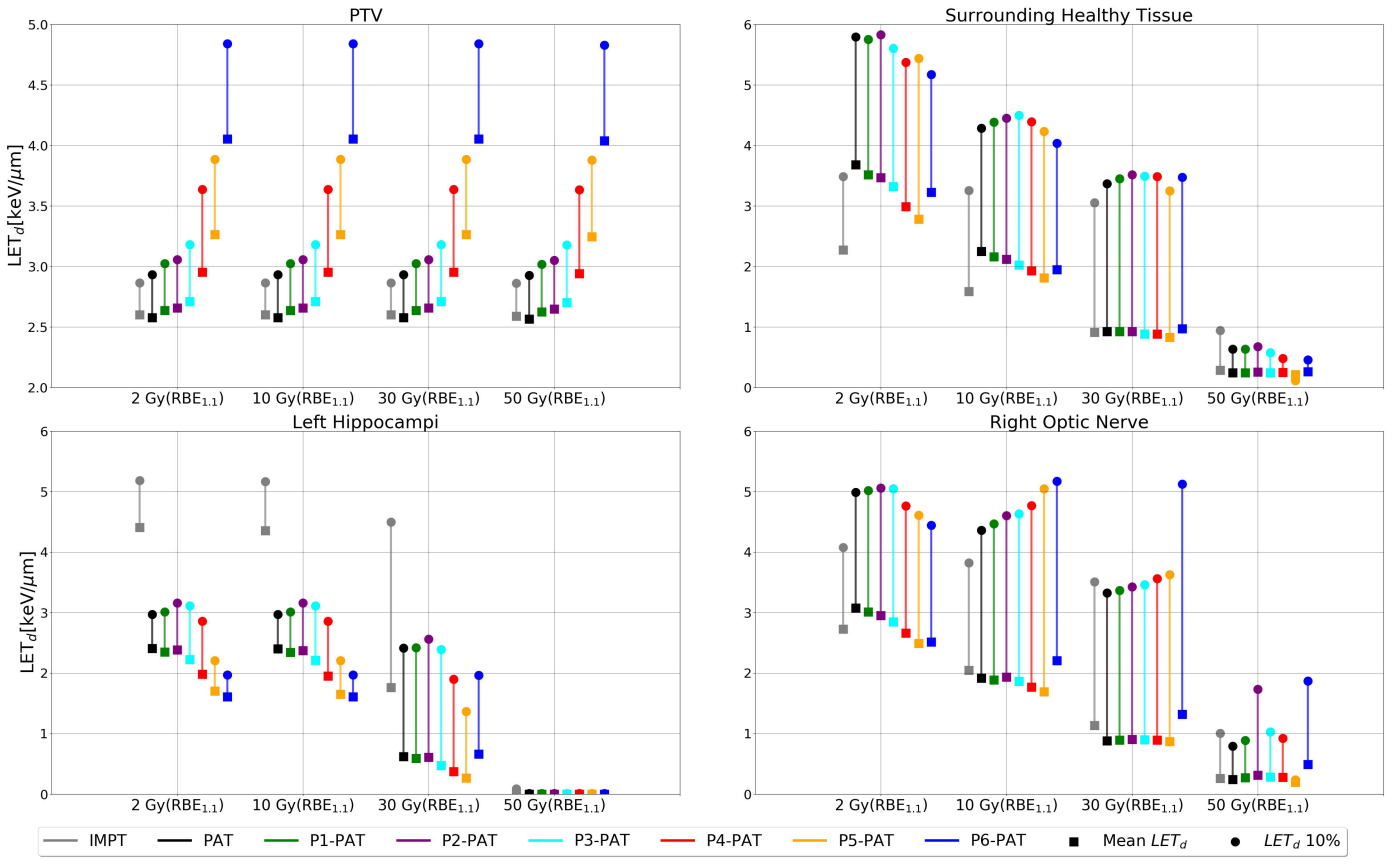
LET_d_ values for the PTV, surrounding healthy tissue and the OARs with different dose cutoff values for the germinoma case. The square markers represent the mean LET_d_ and the circle markers represent the LET_d_ metrics for 10% of the volume.

**Figure 4:**
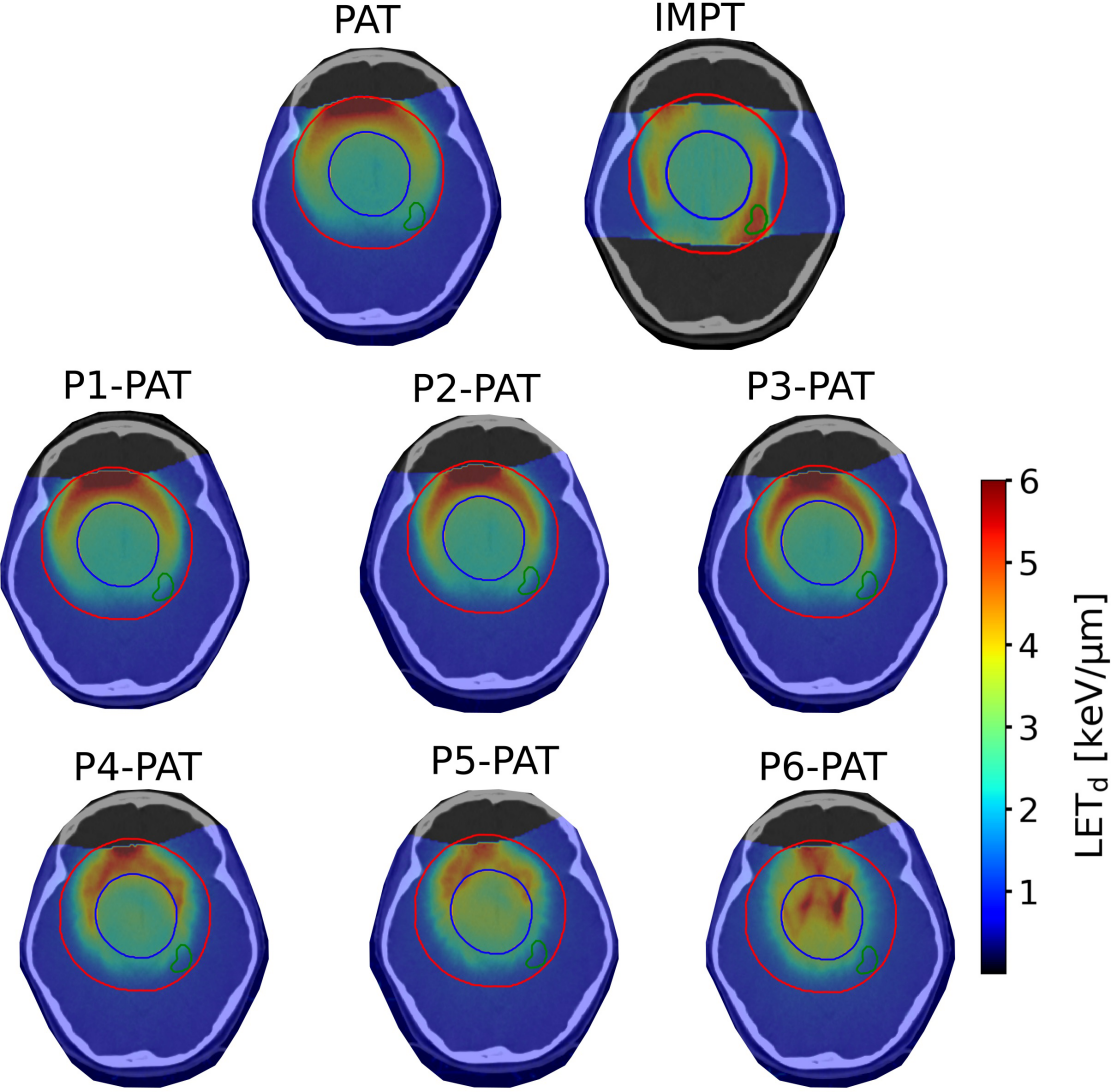
LET_d_ distribution for the different plans with an RBE_1.1_ weighted dose cutoff of 2 Gy(RBE) for the germinoma case. The blue contour represents the PTV, the red contour represents the surrounding healthy tissue, and the left hippocamp is given in green.

For the germinoma case, the P-PAT plans provided higher mean LET_d_ to the target volume, where the P4-, P5-, and P6-PAT plans provided an elevation of respectively 0.4, 0.7 and 1.5. compared to the PAT plan (**Figure 2**). This demonstrates how pruning could effectively increase LET_d_ in the target from the first pruning iteration. The pruning also led to a decrease in mean LET_d_ in the surrounding healthy tissue and the OARs for all the P-PAT plans except for the P6-PAT plan, and this decrease was consistent for all dose cutoff-values (**Figure 3**). Further, for the non-pruned plan a halo of high LET_d_ values surrounded the target volume. This effect decreased with each pruned beam for the P-PAT plans, simultaneously as the LET_d_ became more elevated in the target volume (**Figure 4**). LET_d_-volume histograms for the other cutoff values can be found in **Supplementary Materials** (**Figures A1, A2, A3**) as well as the LET_d_ metrics for 2% of the volume (**Figure A4**).

**Figure 5:**
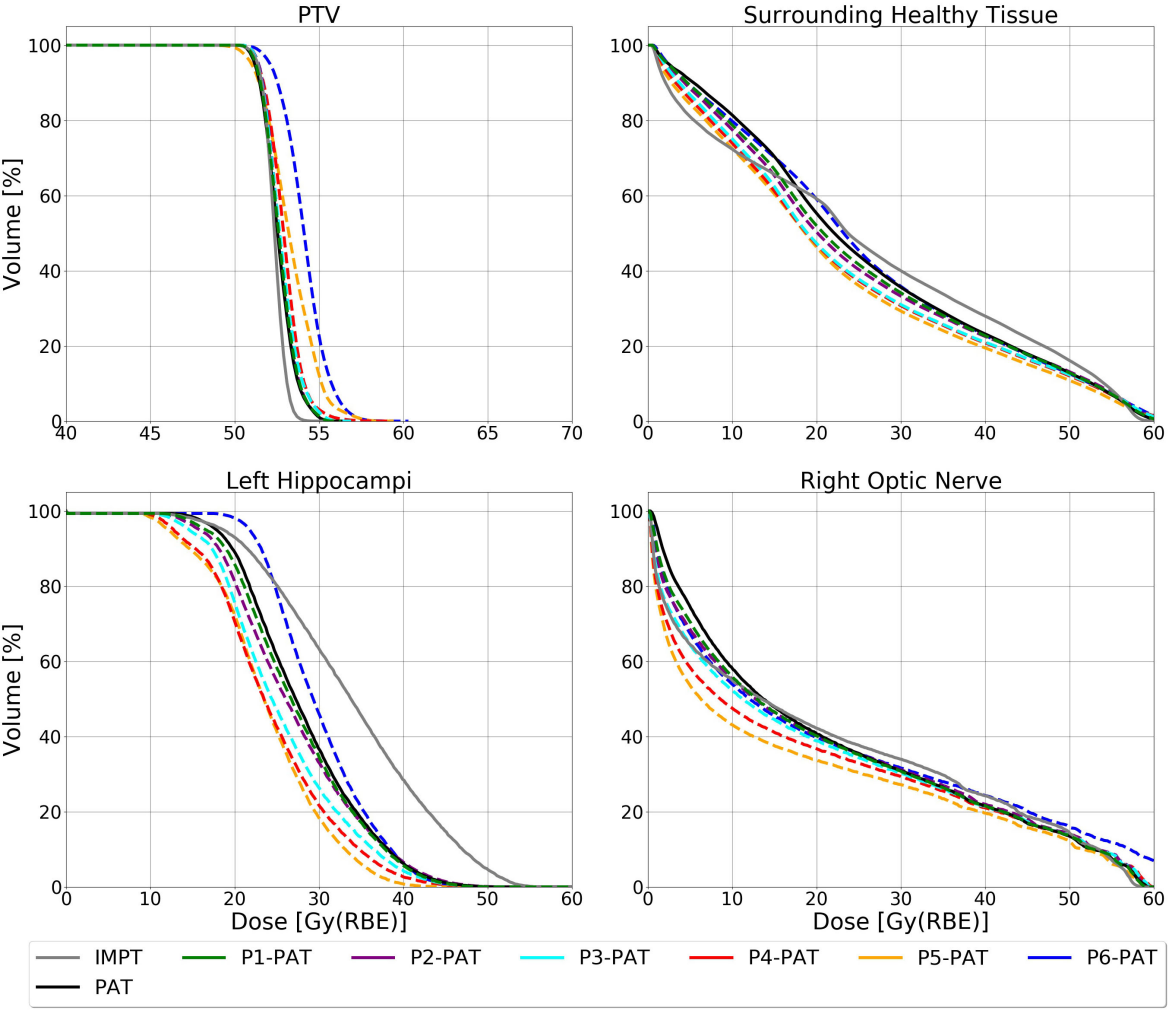
DVHs for the PTV (only showing doses between 40 and 70 Gy(RBE)), surrounding healthy tissue and the OARs calculated with the ROR model for the germinoma case. The dashed lines represent the P-PAT plans, while the solid black and gray line represents the PAT plan and IMPT plan, respectively.

Due to the RBE-LET_d_ dependence, higher RBE-weighted dose from the variable RBE models in the PTV was observed for each pruning iteration (**Figure 5**). The P6-PAT plan provided a considerably higher mean RBE-weighted dose compared to the PAT plan, where the increase in mean RBE-weighted doses were 1.5 Gy(RBE) and 4.0 Gy(RBE) for the ROR model and the LWD model, respectively. The DVH for the LWD model can be found in **Figure A5** in the **Supplementary Materials**. All P-PAT plans were comparable in their ability to meet the planning criteria, with only small variations for the P5-PAT plan as shown in **Figure A7A** in the **Supplementary Materials**.

**Figure 6:**
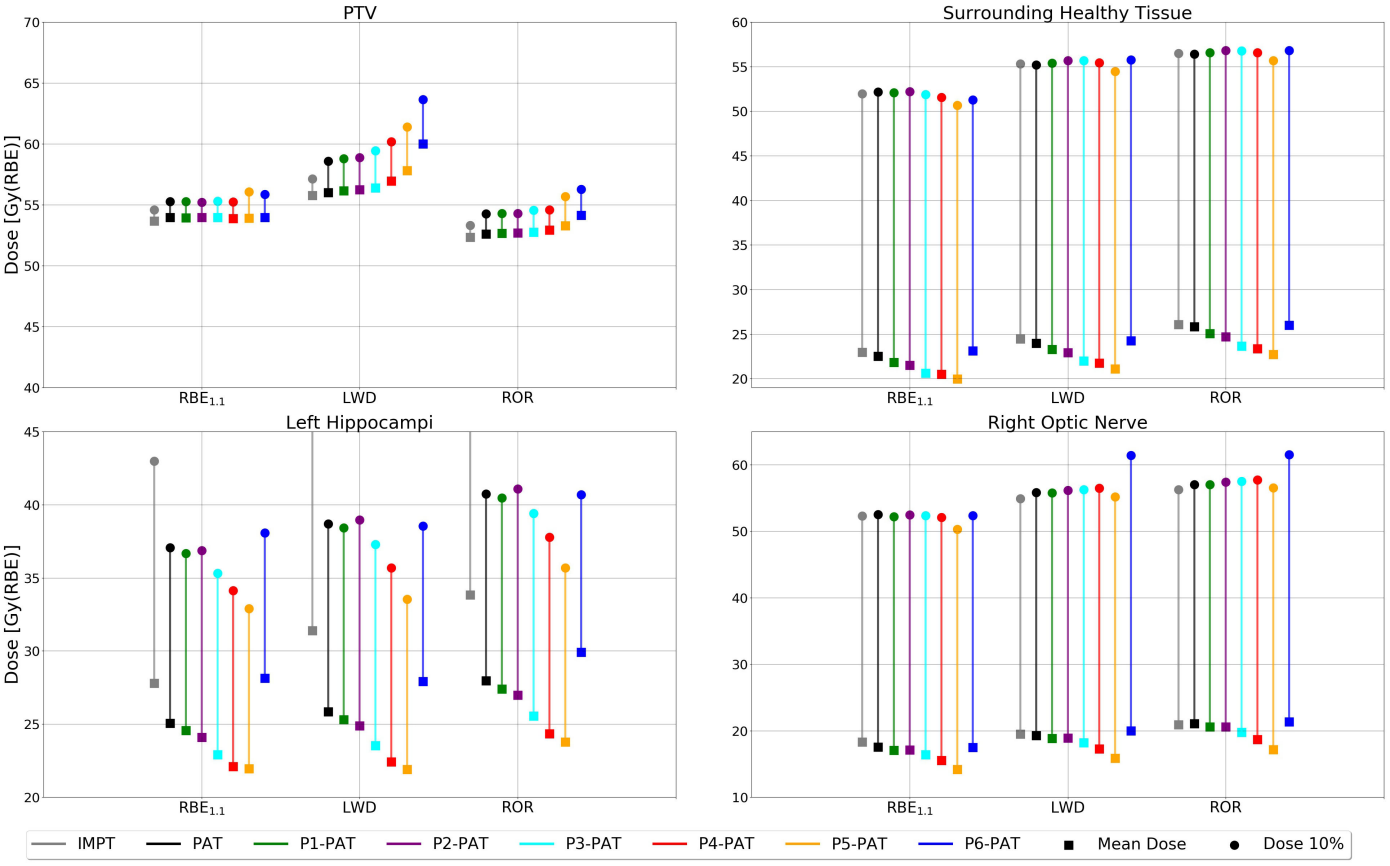
RBE-weighted dose values for the PTV, surrounding healthy tissue and the OARs for the germinoma case calculated with different RBE models. The square marker represents the mean RBE-weighted dose and the circle markers represent the RBE-weighted dose metrics for 10% of the volume.

All P-PAT plans except for the P6-PAT plan reduced the mean RBE-weighted dose to the surrounding healthy tissue and OARs compared to the PAT plan (**Figure 6**). The P6-PAT plan did also give a substantially higher variable RBE-weighted dose to the PTV due to the high LET, and the increase in high RBE weighted dose (10%) in the surrounding healthy tissue was low, as well as the RBE weighted dose for 2% of the volume (**Figure A7** in the **Supplementary Materials**). This was also supported by the integral doses, where a decrease was observed for all pruned plans except the P6-PAT plan (**Figure A8** in the **Supplementary Materials**), which was also consistent for the different RBE models. The lowest mean dose to the surrounding healthy tissue was achieved with the P5-PAT plan, which reduced the mean dose by 2.5 Gy(RBE) compared to the PAT plan, as calculated with an RBE of 1.1 (**Figure 6**). This was also seen in the OARs, where the mean RBE weighted doses were clearly reduced for all P-PAT plans except for the P6-PAT plan (**Figure 6**). This was consistent in the high doses (10%) for the P-PAT plans as well, except for the surrounding healthy tissue, where the dose from the P6-PAT plans showed no increase compared to the PAT plan. The P6-PAT plan would also give opportunity to reduce the physical dose in the surrounding healthy tissue, as the elevated variable RBE-weighted dose to the PTV indicates that lower physical dose is needed to achieve tumor coverage.

When comparing IMPT to PAT, we see clear differences, both in terms of RBE-weighted dose, and LET_d_. The mean LET_d_ in the PTV for the IMPT plan is similar to the PAT plan, with the P4-, P5-, and P6-PAT plans provided an elevation of 0.35, 0.76 and 1.45 keV/μm , compared to the IMPT plan, respectively. The LET_d_ difference in the OARs, however were substantial, as the IMPT plan provided a 2.0 keV/μm increase in the left hippocampus and a 0.9 keV/μm reduction in the left optic nerve, with a 2 Gy(RBE) cutoff. These differences were smaller for higher cutoff values.

We also saw a higher RBE-weighted dose to the surrounding healthy tissue for the PAT plan compared to the IMPT (**Figure 6**). However, this is due to the increased physical dose, where the IMPT plan provided 2.24 and 5.78 Gy(RBE) higher RBE1.1-weighted dose to the left hippocampus compared to the PAT plan and P5-PAT plan, respectively.

### 3.2 Ependymoma

**Figure 7:**
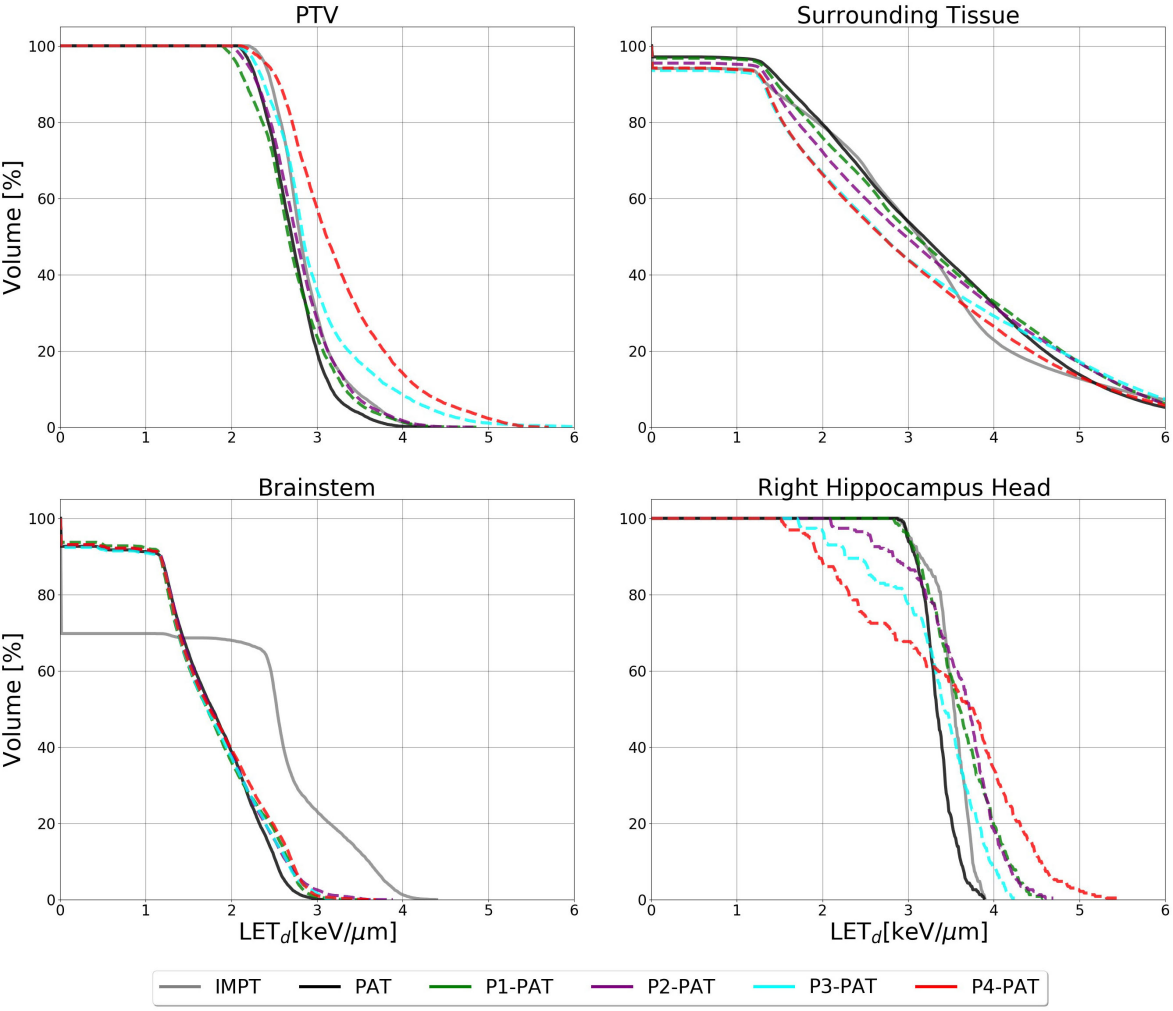
LET_d_ volume histogram for the PTV, surrounding healthy tissue and the OARs calculated with 2 Gy(RBE_1.1_) dose cutoffs for the ependymoma case. The dashed lines represent the P-PAT plans, while the solid black and gray lines represent the PAT and IMPT plans, respectively.

For the ependymoma case, we saw similar results as with the germinoma case, i.e. plans with high level of pruning achieved the highest LET_d_ in the PTV. The mean LET_d_ for the P2, P3 and P4-PAT plans provided an elevation of 0.27, 0.25 and 0.53 keV/μm compared to the PAT plan, respectively (**Figure 7**). There was also a clear LET_d_ reduction in the surrounding healthy tissue for the P3- and P4- PAT plans, as compared to the reference PAT plan. For the OARs, however, the P-PAT plans provided a similar or higher LET_d_ compared to the PAT plan.

**Figure 8:**
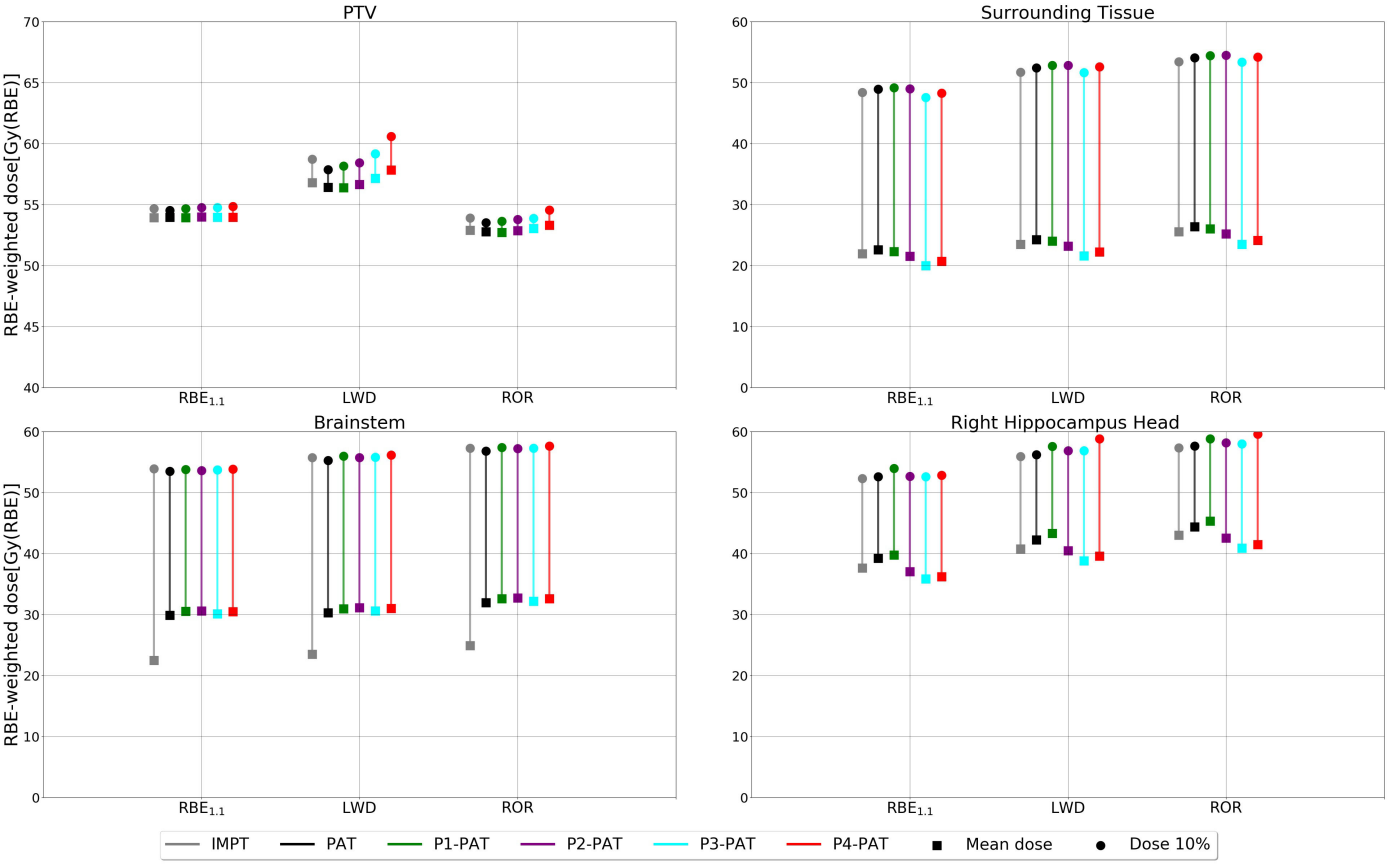
RBE-weighted dose values for the PTV, surrounding healthy tissue and the OARs for the ependymoma case, calculated with different RBE models. The square marker represents the mean RBE-weighted dose and the circle markers represent the RBE-weighted dose metrics for 10% of the volume.

The metrics in **Figure 8** shows that for the P-PAT plans, the mean RBE-weighted dose is reduced in the surrounding healthy tissue for all RBE models, compared to the reference PAT plan, due to the lower corresponding RBE1.1 -weighted dose. This reduction was also seen in mean RBE-weighted dose to the right hippocampal head except the P1-PAT plan. Additional results for the ependymoma case can be found in the **Supplementary Materials**.

For this case we also see a lower RBE1.1-weighted dose for the IMPT plan compared to the PAT plan in the surrounding tissue and the OARs. The IMPT plan provided a 0.65 Gy(RBE) lower mean RBE1.1 in the brainstem compared to the PAT plan.

### 3.3 Rhabdomyosarcoma

**Figure 9:**
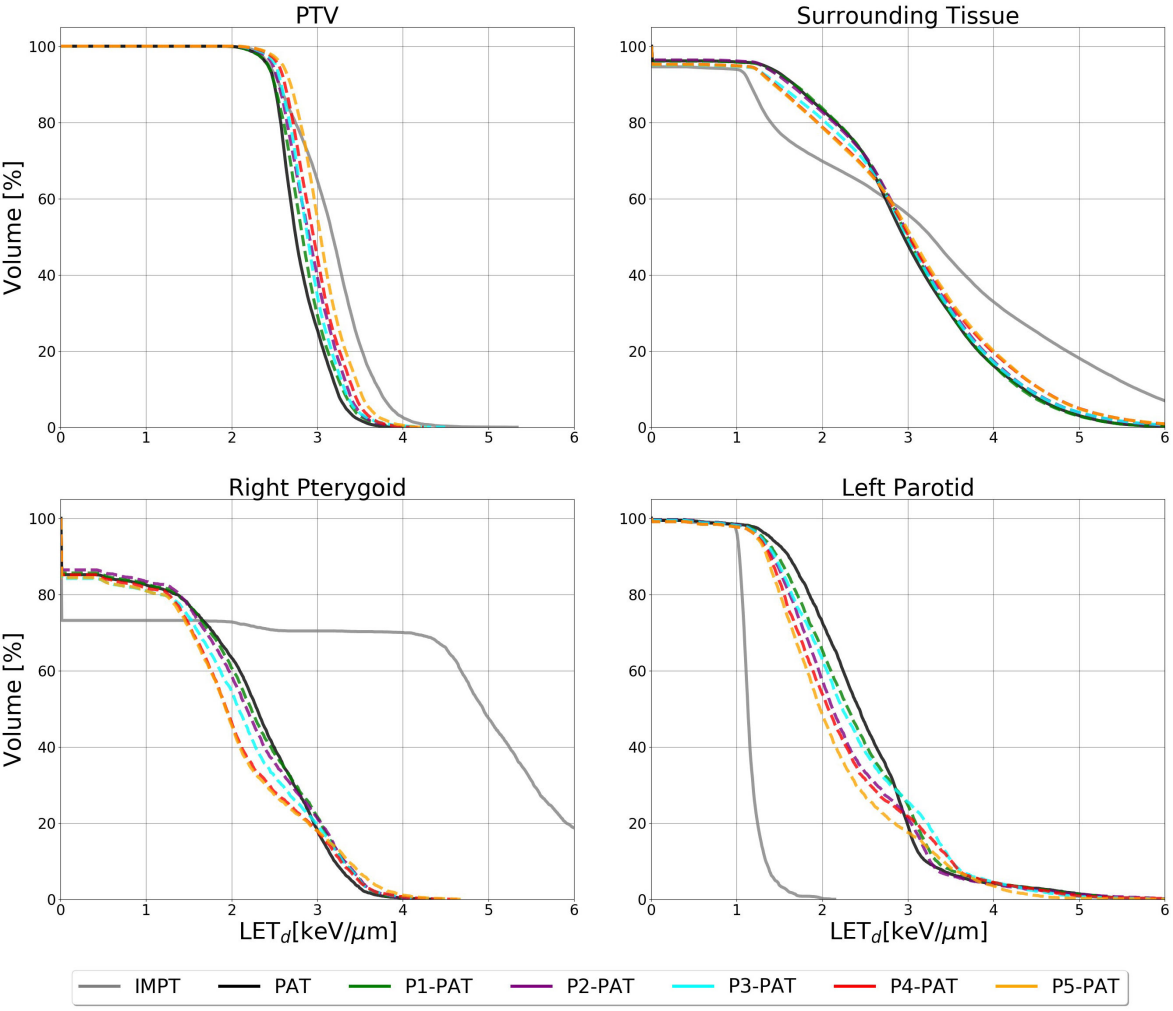
LET_d_ volume histogram for the PTV, surrounding healthy tissue and the OARs calculated with 2 Gy(RBE1.1) dose cutoffs for the rhabdomyosarcoma case. The dashed lines represent the P-PAT plans, while the solid black and gray lines represent the PAT plan and IMPT plan, respectively.

For the rhabdomyosarcoma case, we continue to see an increase in the LET_d_ in the PTV for the plans with the highest degree of pruning, compared to the PAT plan (**Figure 9**). However, this increase is smaller compared to the germinoma and ependymoma case, as the mean LET_d_ for the P5-PAT plan was 0.25 keV/μm higher than for the PAT-plan. The LETd to the PTV was higher for the IMPT plan compared to the PAT plan, with an increase in mean LETd of 0.35 keV/μm (**Figure 9**).We also saw a small decrease in the LET_d_ to the surrounding healthy tissue and OARs for the P-PAT plans as compared to the PAT plans, but this difference was smaller compared to the other cases.

**Figure 10:**
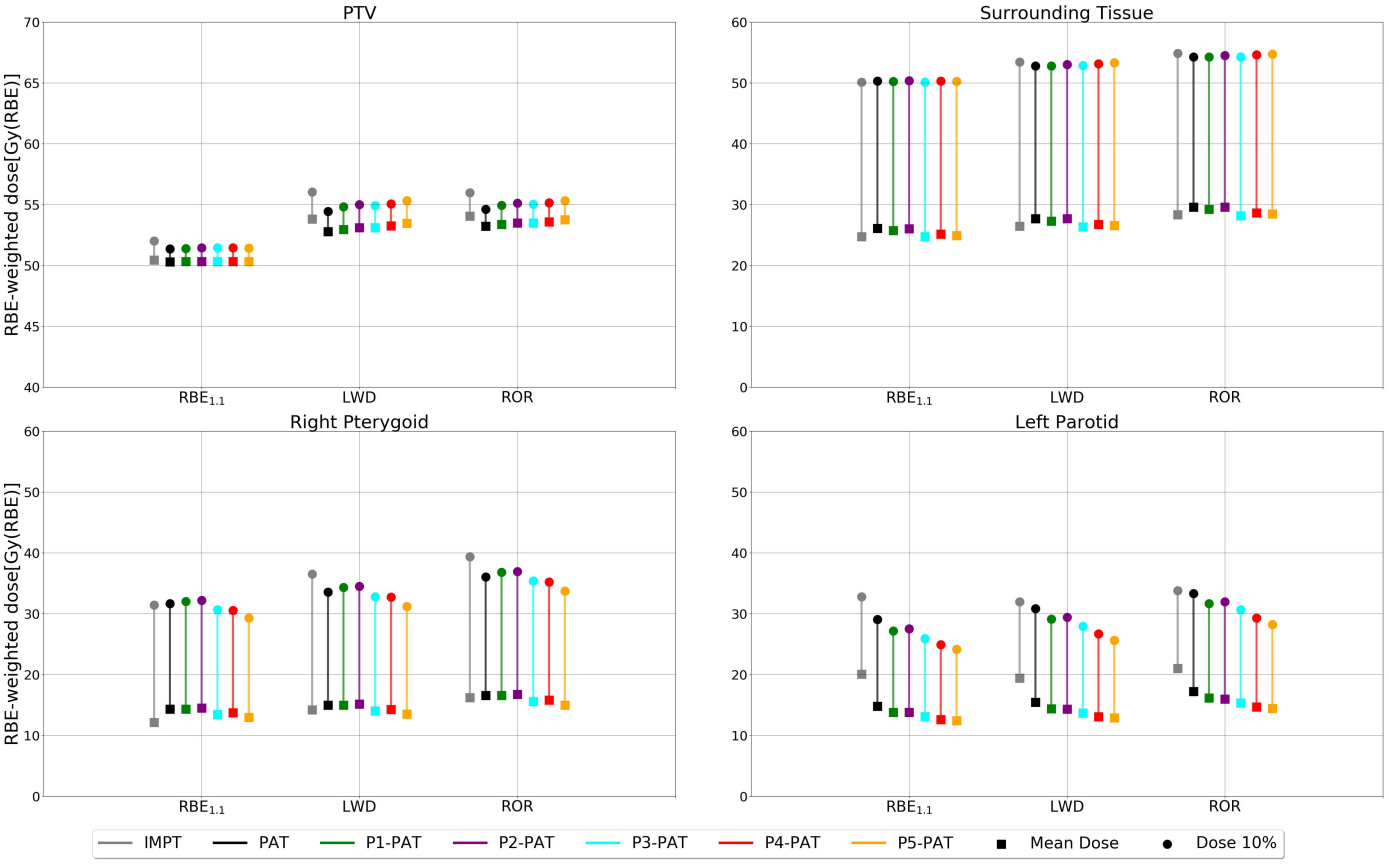
RBE-weighted dose values for the PTV, surrounding healthy tissue and the OARs for the rhabdomyosarcoma case, calculated with different RBE models. The square marker represents the mean RBE-weighted dose and the circle markers represent the RBE-weighted dose metrics for 10% of the volume.

There is, however, a clear decrease in both the mean and max (D10%) RBE-weighted dose for the P-PAT plans in both OARs for all RBE models (**Figure 10**). The RBE-weighted dose in the PTV was also higher for the variable RBE models compared to the plan recalculated with RBE_1.1_, with a mean dose of 2.96 and 2.48 Gy(RBE) for the PAT plans recalculated with the ROR and LWD model, respectively. Compared to the PAT plan, the mean RBE1.1-weighted dose to the left parotid and right pterygoid were 5.2 Gy(RBE) higher and 2.2 Gy(RBE) lower for IMPT, respectively. This is an indication that the tumor location is an important parameter when comparing PAT and IMPT. Additional results for the rhabdomyosarcoma case can be found in the **Supplementary Material**.

## 4 Discussion

Emerging clinical evidence of protons having increased biological effect at the distal end of the beam motivated the method for pruning PAT plans in order to avoid high LET_d_ values surrounding the target volume. Pruned PAT plans for three different cases with adequate target volume coverage were found to have increased LET_d_ in the target volume for each degree of pruning and corresponding decrease in LET_d_ in the healthy tissue surrounding the target. Despite the inability of each beam in the P-PAT plans to cover the target volume completely, coverage similar to the reference PAT-plan was achieved for each P-PAT plan.

The physical dose was slightly increased in the surrounding healthy tissue for the P6-PAT germinoma plan compared to the other pruned plans and the IMPT plan. Consequently, the RBE-weighted dose was also increased when considering variable RBE and the reduced LET outside the target in the P6-PAT plan. Similar reduction in LET_d_ in normal tissue, and increase in target has been seen in studies using monoenergetic arcs which, in addition to saving treatment time (46), could also provide a more desirable LET_d_ distribution (35, 36). The elevated RBE-weighted dose in the surrounding healthy tissue could be an argument for avoiding excessive pruning and a balance between improved LET_d_ distribution and potential unfavorable dose distribution must be considered in the pruning process. This is possibly a consequence of increased proximal dose for each individual beam. Additionally, the pruning technique is only demonstrated on three patient cases, and the optimal degree of pruning may vary depending on the tumor site and anatomy, further than what we saw in this study. More case studies are therefore needed for improvements and generalization of this technique. However, the number of beams and the tumor size did vary between the cases, and consistent elevation of the LET_d_ in the PTV was observed. A different beam setup could also improve the LET and RBE distribution e.g., two half arcs of 120 degrees. We also saw that the surrounding halo of high LET_d_ values around the PTV shrunk for each pruning iteration, while the LET_d_ increased in the tumor volume. This technique of pruning could therefore have clinical benefits, as several recent studies have shown strong correlation between radiation induced image changes and LET_d_ (10–21). Especially the peri-ventricular system is at an increased risk with higher RBE and LET (10). We also saw a reduction in the number of spots per pruning iteration, except for the P6-PAT plan, and while the number of spots is still relatively high the beam delivery time can be reduced with the pruning technique. This is also a topic in other studies, where the number of spots is reduced to reduce treatment time (47), achieve improved RBE (48) or reduce risk of toxicity (49).

There is a consensus in proton therapy that the RBE is not constant, but variable and dependent on parameters such as dose, LET_d_ and tissue type (50). The clinically applied RBE of 1.1 is considered a conservative value to ensure tumor control, which raises the question of how variable RBE and LET should be implemented in proton therapy (51). Although there is no agreement on which RBE model gives the most precise description of the variable proton RBE, all models provide a similar shape of the RBE distribution based on the LET, although with a varying magnitude (32). This variation, coupled with the uncertainty of the model input parameters, leads to a reluctance of using variable RBE models in a clinical setting (52). An intermediate stage before clinical implementation of these models is an LET_d_ and RBE evaluation approach, as demonstrated in this study, as well as previous literature (34, 51, 53, 54). This means that clinical plans are optimized with respect to an RBE of 1.1, but other parameters are changed to improve the RBE-weighted dose and LET_d_ distributions. This would give a new dimension in the treatment planning process, as the evaluation of the LET_d_ and RBE can be used to select a more optimal plan. Based on the LET_d_ and RBE-weighted dose distributions in this study, a P-PAT plan could provide a better biological outcome compared to a PAT plan, while still providing similar target volume coverage.

PAT plans have proved their advantage compared to IMPT in terms of conformity and robustness through multiple retrospective treatment planning studies (2, 8, 55). Consequentially, challenging cases with IMPT could be referred to and improved through the introduction of PAT. Brain tumor cases may be particularly relevant since only short proton ranges are needed (1). We also saw a reduced RBE-weighted dose in the healthy surrounding tissue and integral dose for the pruned PAT plans, except for P6-PAT, although beams with lower energies will provide a higher entrance dose.

In this study, two variable RBE models were used, a purely LET_d_ based model (LWD) and a model based on *in vitro* data (ROR), alongside the reference RBE of 1.1. The LWD model is based on a scaling parameter *c*, which in this study was set to 0.055 μm/keV , as previously suggested by McMahon et al. (44) for reduced RBE variability as it was based on a fitting of *in vitro* data. This value is higher than other studies (40, 45, 54), which is reflected in the recalculation from the LWD model which showed a considerable increase in RBE-weighted dose in both the PTV and healthy tissue, suggesting an overdosage of the target volume when including LWD in the dose calculation. The ROR model, on the other hand, has an inverse ${\left(\alpha /\beta \right)}_{x}$ dependency, with the RBE decreases with an increasing ${\left(\alpha /\beta \right)}_{x}$ , which separates it from the LWD model. Since the ${\left(\alpha /\beta \right)}_{x}$ for a germinoma tumor volume is high, while the LET_d_ in the tumor is relatively low for the PAT plan and the P1-, P2- and P3-PAT plans, we did not see the same increase in RBE-weighted dose as with the LWD model, with the ROR model giving a lower RBE-weighted tumor dose compared to the RBE_1.1_ model. With a lower tumor ${\left(\alpha /\beta \right)}_{x}$ , the P-PAT plans would provide a higher effect for the ROR model. This was seen for the rhabdomyosarcoma case, as well as in in the normal tissue for both the germinoma and ependymoma case, as similar results between the LWD and ROR were found, illustrating the strong ${\left(\alpha /\beta \right)}_{x}$ dependency of ROR.

Although the difference between these models illustrates the uncertainties that come with variable RBE models, it can also demonstrate a method for choosing a treatment plan if the clinical objectives are met. The reduction in RBE-weighted dose and LET_d_ in the healthy tissue for the P3, P4 and P5-PAT plans for the ependymoma and the rhabdomyosarcoma case, suggests that they could provide a better treatment outcome compared to a regular PAT plan, despite the further increase in RBE-weighted dose to the surrounding tissue from the highest pruned P-PAT plan.

Although P-PAT showed an improved LET_d_ distribution, this is a blind approach for optimizing the LET_d_, as long as commercial TPS’s do not have methods for calculating it, although this is expected in newer versions. However, as recommended by Indelicato et al. (56), no more than a third of proton beams should end in brainstem tissue outside the PTV, due to the high LET values that occur in the distal end. This is also a blind approach for accounting for LET_d_, similar to what we explore in this study. LET calculation and optimization is a planned upgrade for some proton TPSs. Pruning techniques could potentially also be a valuable tool for guiding the optimizers and providing knowledge for improved LET-distributions.

## 5 Conclusion

In this study we demonstrated how pruning techniques in PAT can contribute to higher LET_d_ values in the target volume and simultaneously reduce the LET_d_ in surrounding healthy tissue. We showed that by evaluating the LET_d_ and the RBE-weighted dose from P-PAT plans, a more optimal plan based on RBE-weighted dose can be constructed without compromising target volume coverage or increasing OAR dose.

## Data availability statement

The raw data supporting the conclusions of this article will be made available by the authors, without undue reservation.

## Author contributions

All corresponding authors have contributed directly to the intellectual content of this manuscript HH, SP, LF, KSYH and CHS suggested the idea for this study and provided the basis for the method. HH was responsible for performing the method, analyzing the data, and is the main author. All authors have contributed to the discussion of the results All authors have read and approved this manuscript.
