# Supplementary material for: Influence of beam pruning techniques on LET and RBE in proton arc therapy
Source: Front Oncol. 2023 Sep 5;13:1155310. doi: 10.3389/fonc.2023.1155310 (PMC10508957; doi:10.3389/fonc.2023.1155310)
Supplement: Supplementary file 1 [file DataSheet_1.docx]

### **Additional results for the germinoma case**

In Figure A1-Figure A3, we see the LET_d_ volume histograms for the different cutoff-values. In Figure A5 and Figure A6 we see the DVHs for the LWD model and the RBE_1.1_ model, respectively, while Figure A4 and Figure A7 show additional scatterplots for LET_d_ and RBE weighted dose, respectively. Figure A8 show the integral doses for the different RBE models.


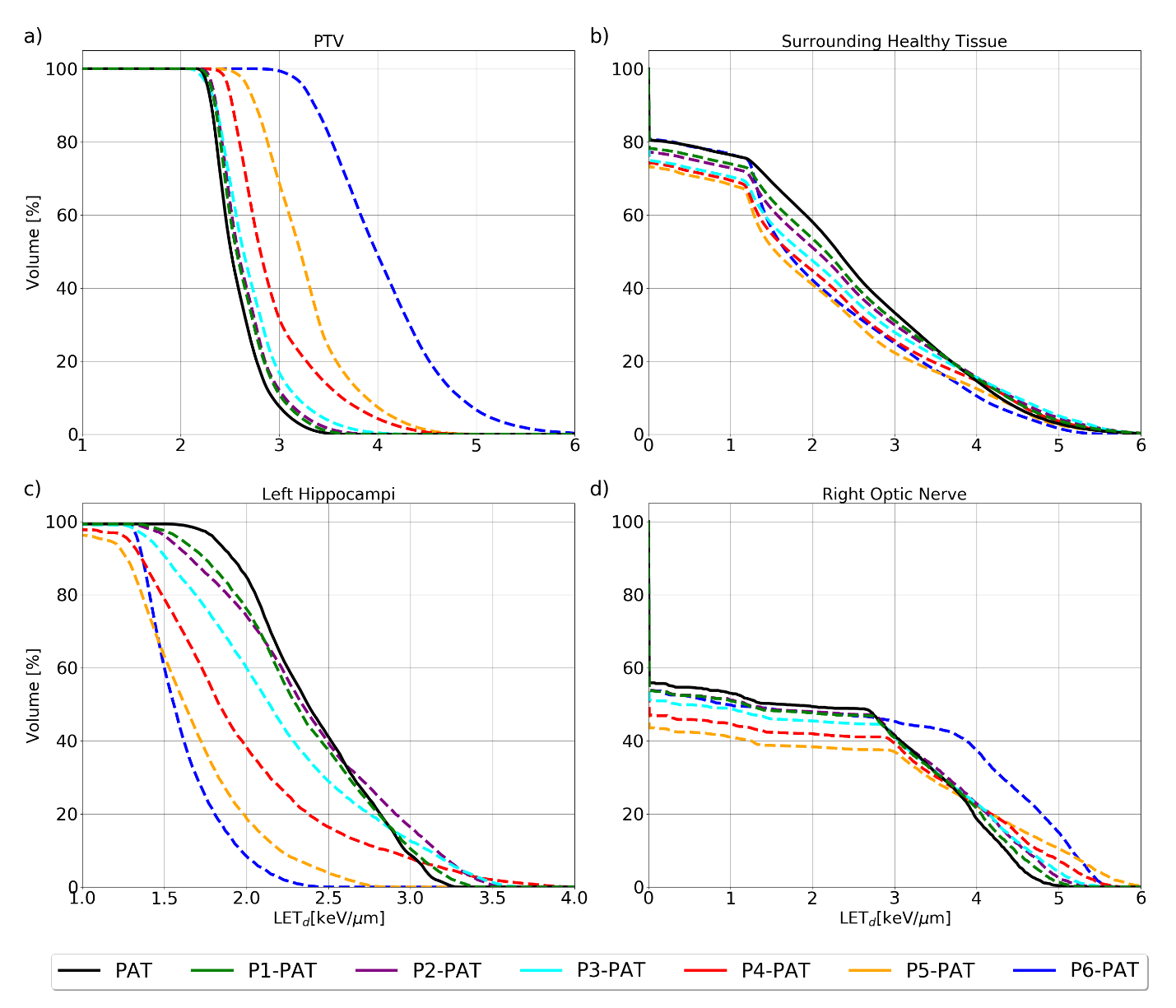


Figure A1 LET_d_ volume histogram for the PTV and OARs calculated with 10 Gy(RBE_1.1_) dose cutoffs for the germinoma case. The dashed lines represent the P-PAT plans, while the solid lines represent the PAT plan.


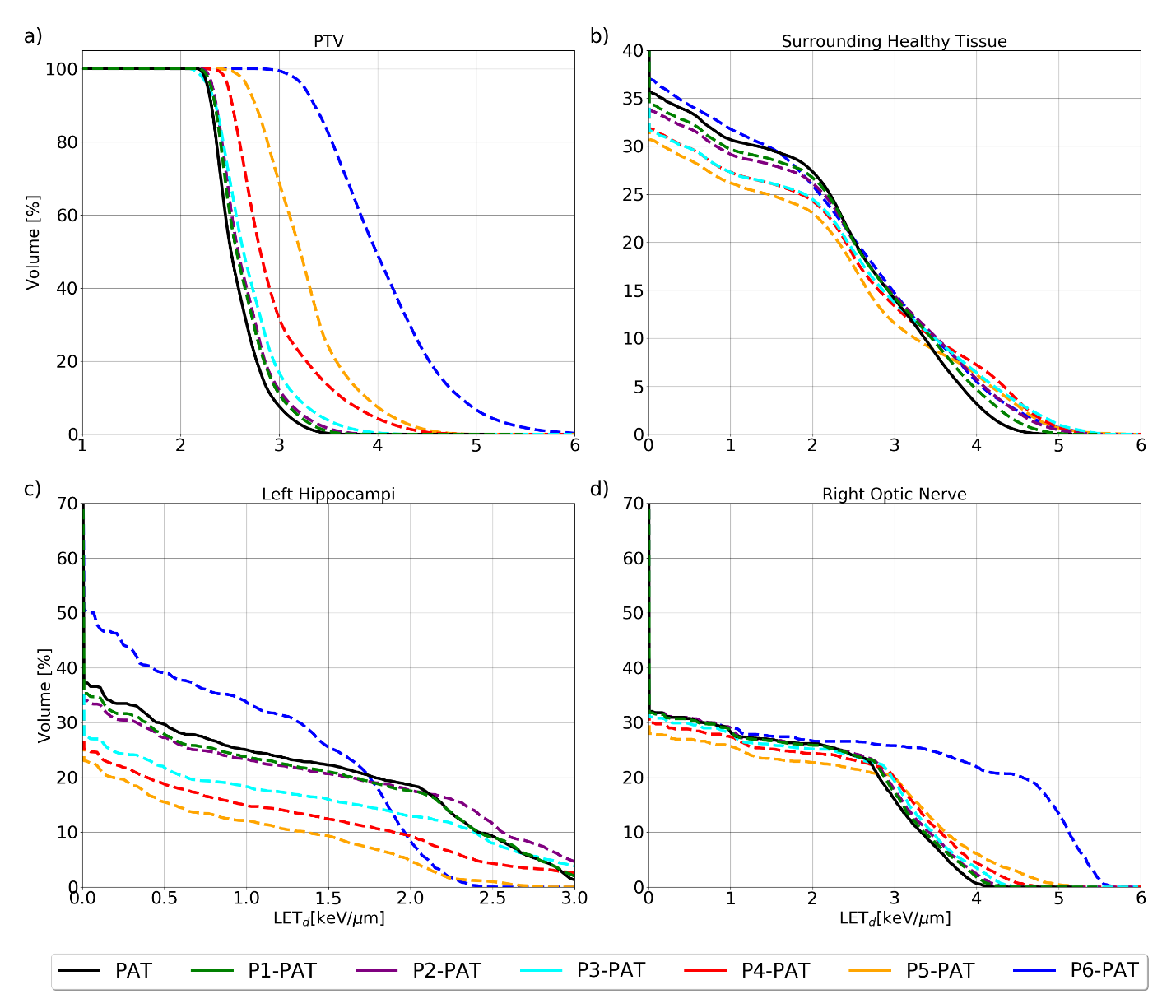


Figure A2 LET_d_ volume histogram for the PTV and OARs calculated with 30 Gy(RBE_1.1_) dose cutoffs for the germinoma case. The dashed lines represent the P-PAT plans, while the solid lines represent the PAT plan.


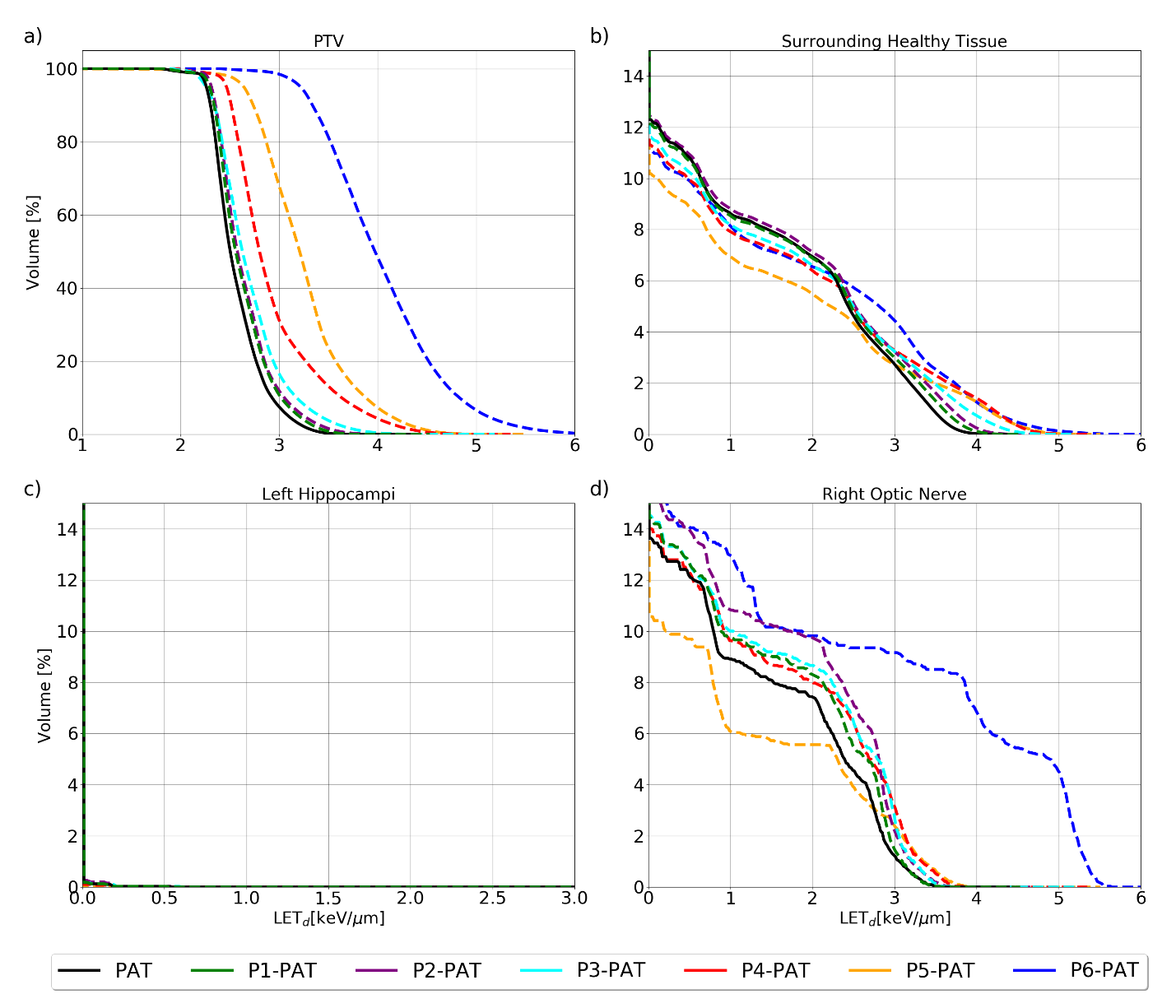


Figure A3 LET_d_ volume histogram for the PTV and OARs calculated with 50 Gy(RBE_1.1_) dose cutoffs for the germinoma case. The dashed lines represent the P-PAT plans, while the solid lines represent the PAT plan.


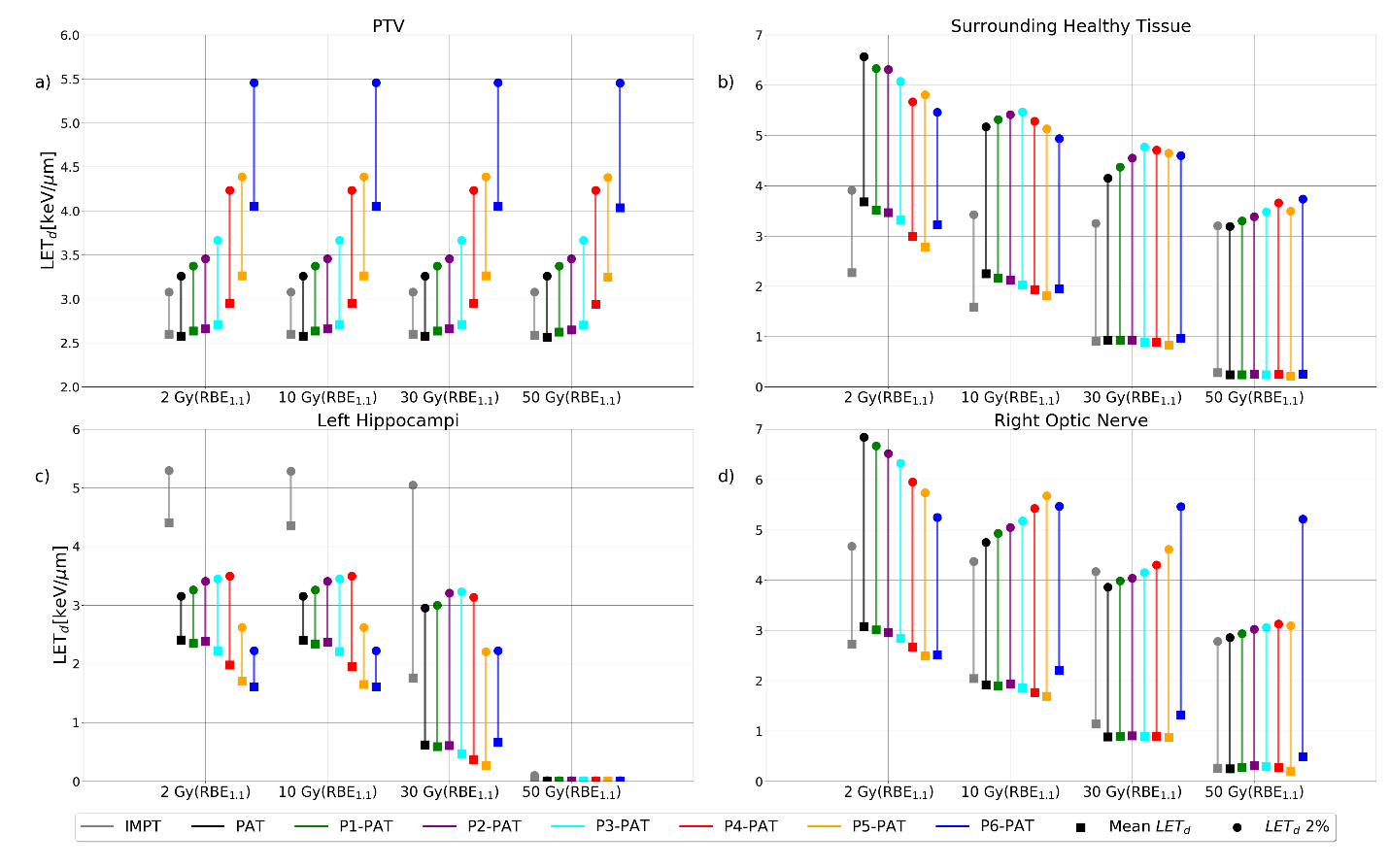


Figure A4 LET_d_ values for the PTV, surrounding healthy tissue and the OARs with different dose cutoff values for the germinoma case. The square markers represent the mean LET_d_ and the circle markers represent the LET_d_ metrics for 2% of the volume.


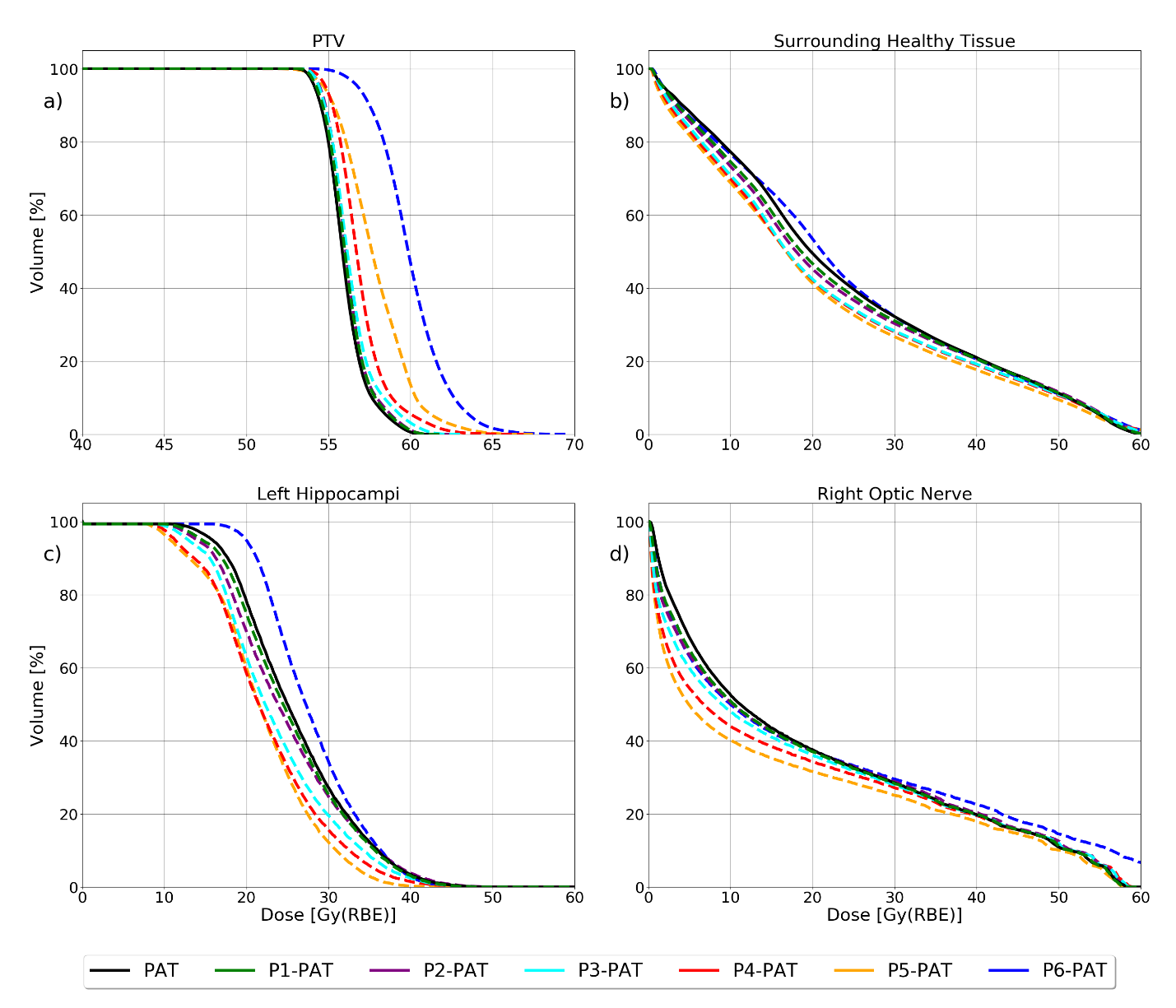


Figure A5 DVH for the LWD plan for the PTV, surrounding healthy tissue and the OARs for the germinoma case. The dose from the PAT plan is represented by solid lines, while the P-PAT plans are represented with dashed lines.


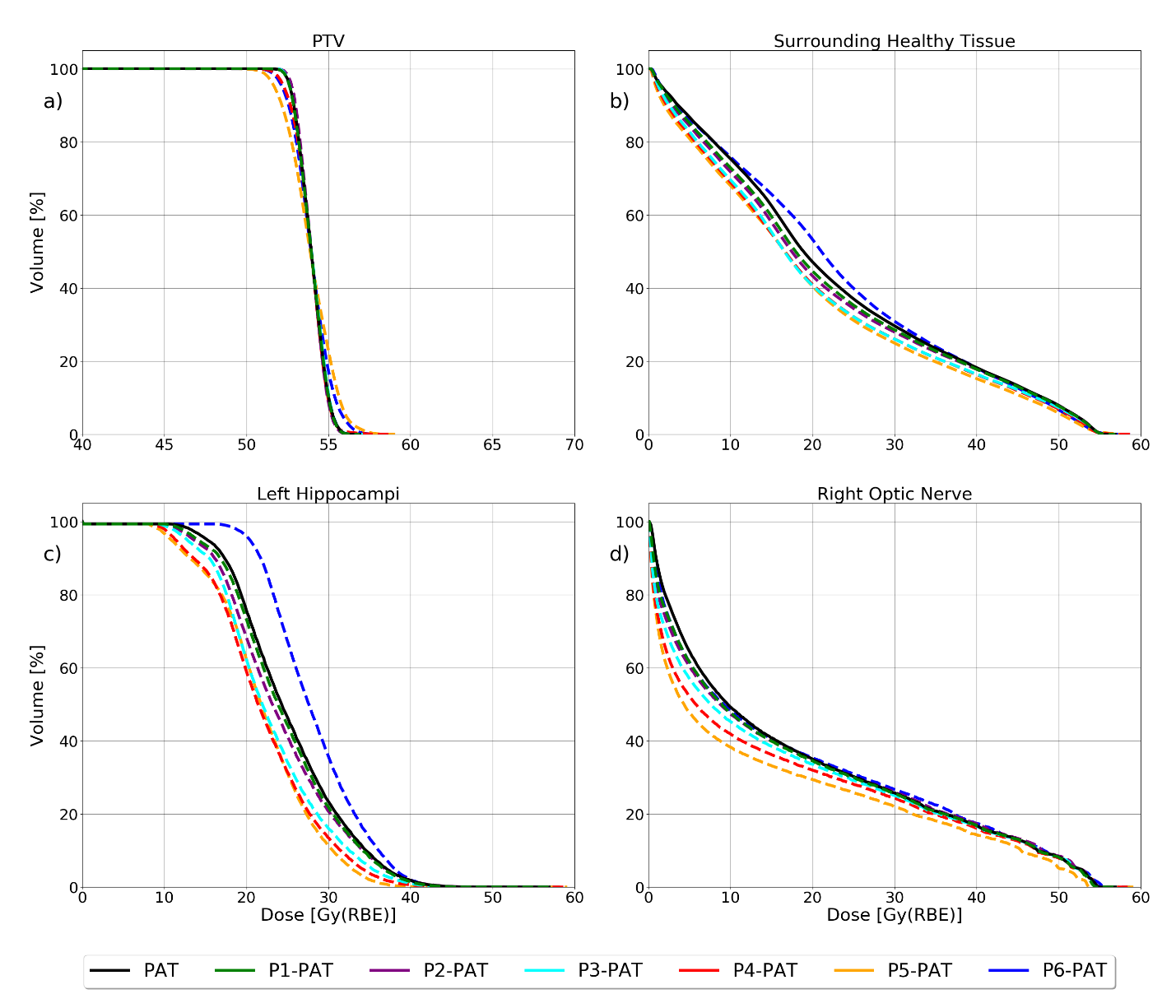


Figure A6 DVH for the RBE_1.1_ plan for the PTV, surrounding healthy tissue and the OARs for the germinoma case. The dose from the PAT plan is represented by solid lines, while the P-PAT plans are represented with dashed lines.


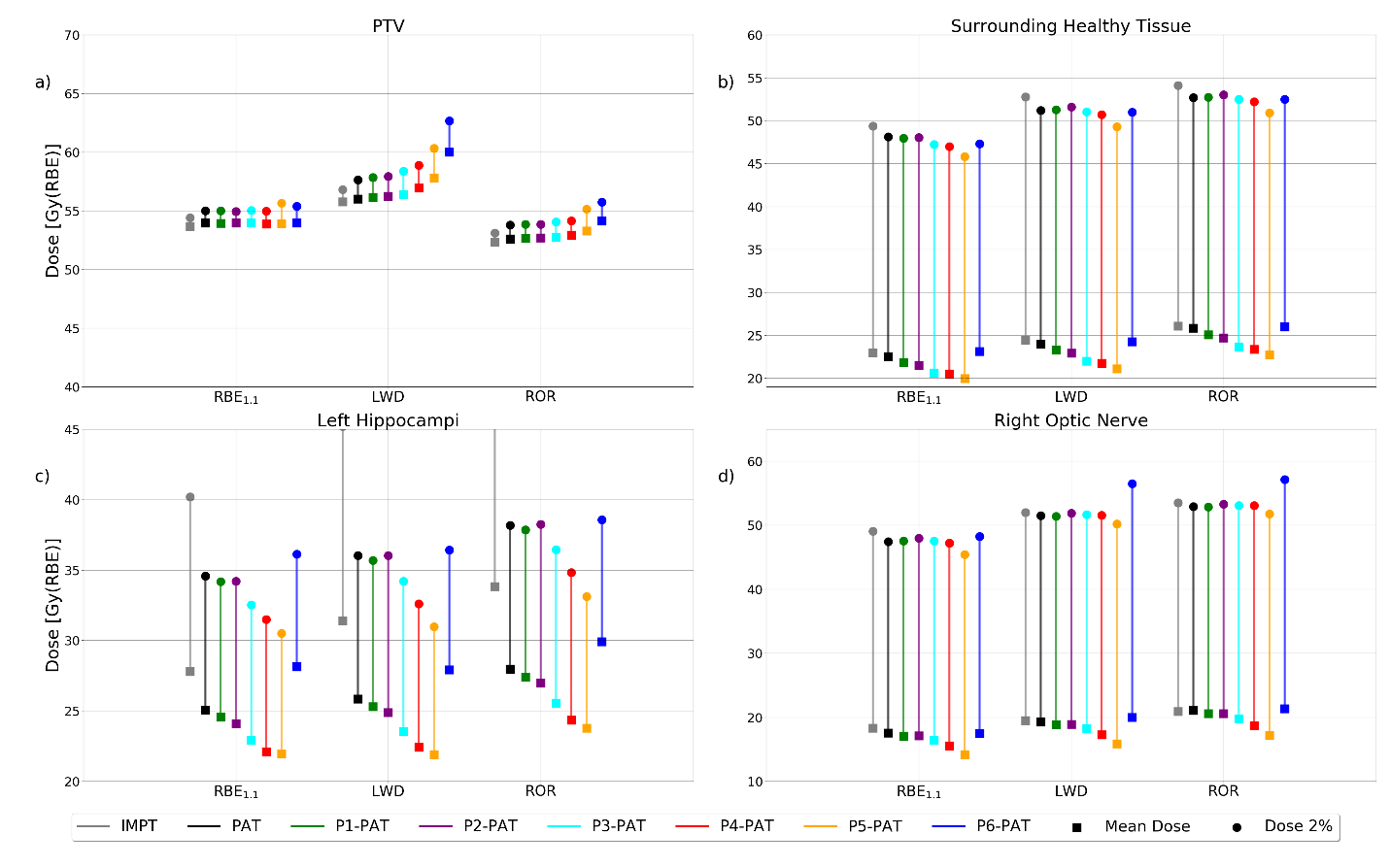


Figure A7 RBE-weighted dose values for the PTV, surrounding healthy tissue and the OARs for different RBE models for the germinoma case. The square marker represents the mean RBE-weighted dose and the circle markers represent the RBE-weighted dose metrics for 2% of the volume.


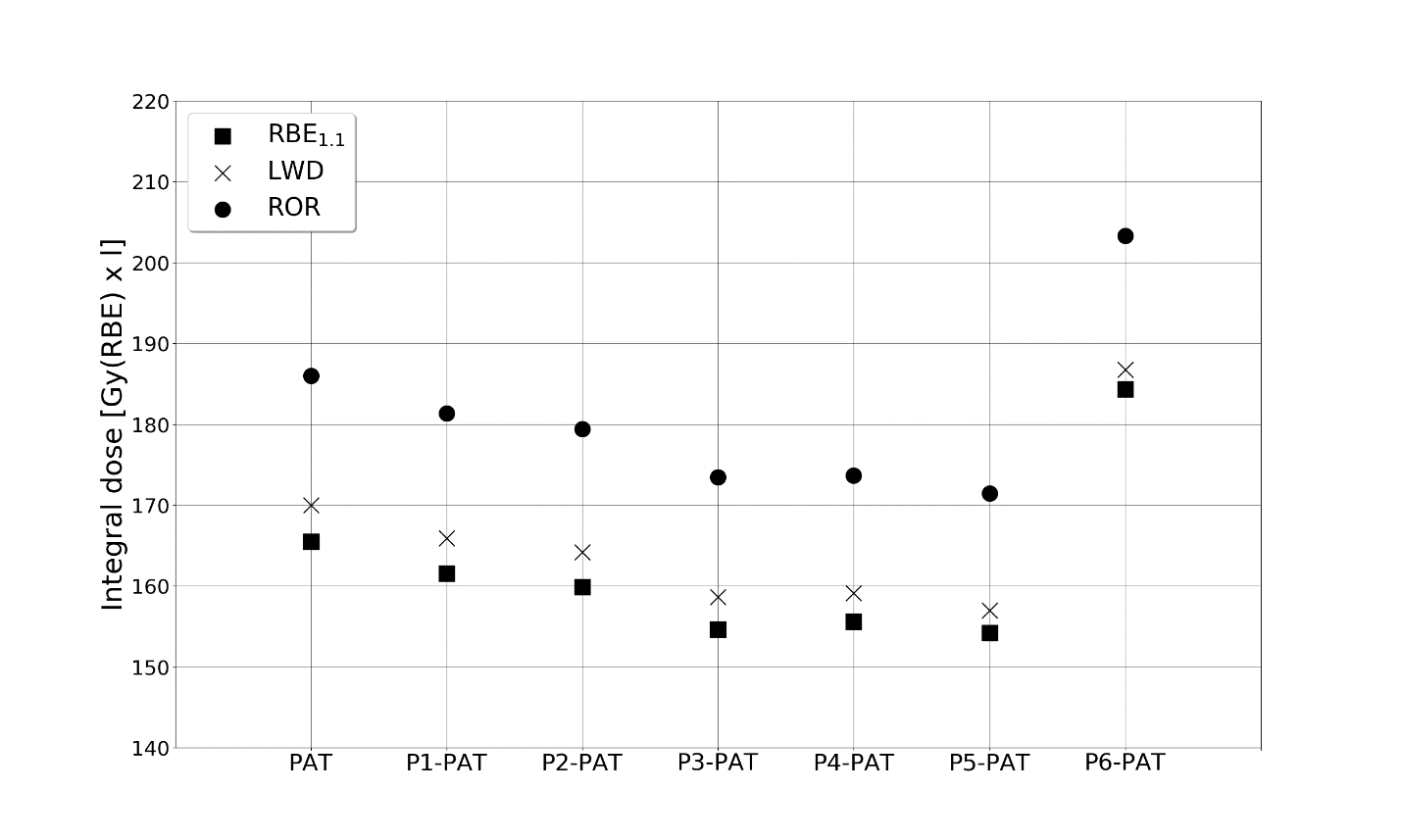


Figure A8 Integral doses for the different plans, where the different markers represent the different RBE-models for the germinoma case. The integral doses are calculated as the total dose to a volume times the volume.

### **Additional results for the ependymoma case**

In Figure A9, Figure A10, and Figure A11, we see the LET_d_ volume histograms for the different dose cutoff-values, and in Figure A12, a colorwash of the LET_d_ for the different P-PAT plans are given. In Figure A13 and Figure A14 we see a scatterplot for the LET_d_ for different metrics. DVHs for the different RBE-models are given in Figure A15, Figure A16 and Figure A17, while Figure A18 show an additional scatterplot for the RBE weighted dose metrics. Figure A19 shows the integral dose for all RBE-models.


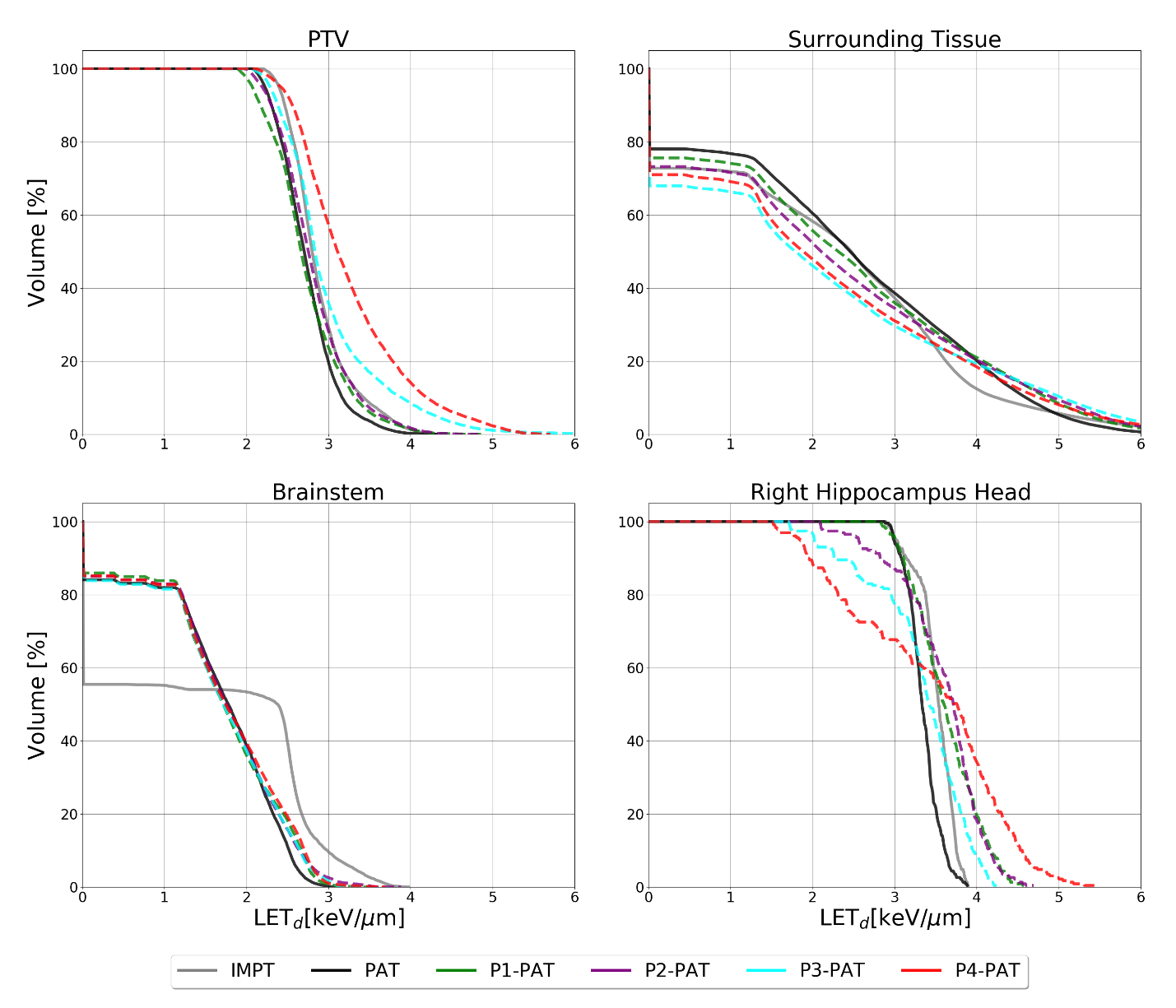


Figure A9 LET_d_ volume histogram for the PTV and OARs calculated with 10 Gy(RBE_1.1_) dose cutoffs for the ependymoma case. The dashed lines represent the P-PAT plans, while the solid lines represent the PAT plan.


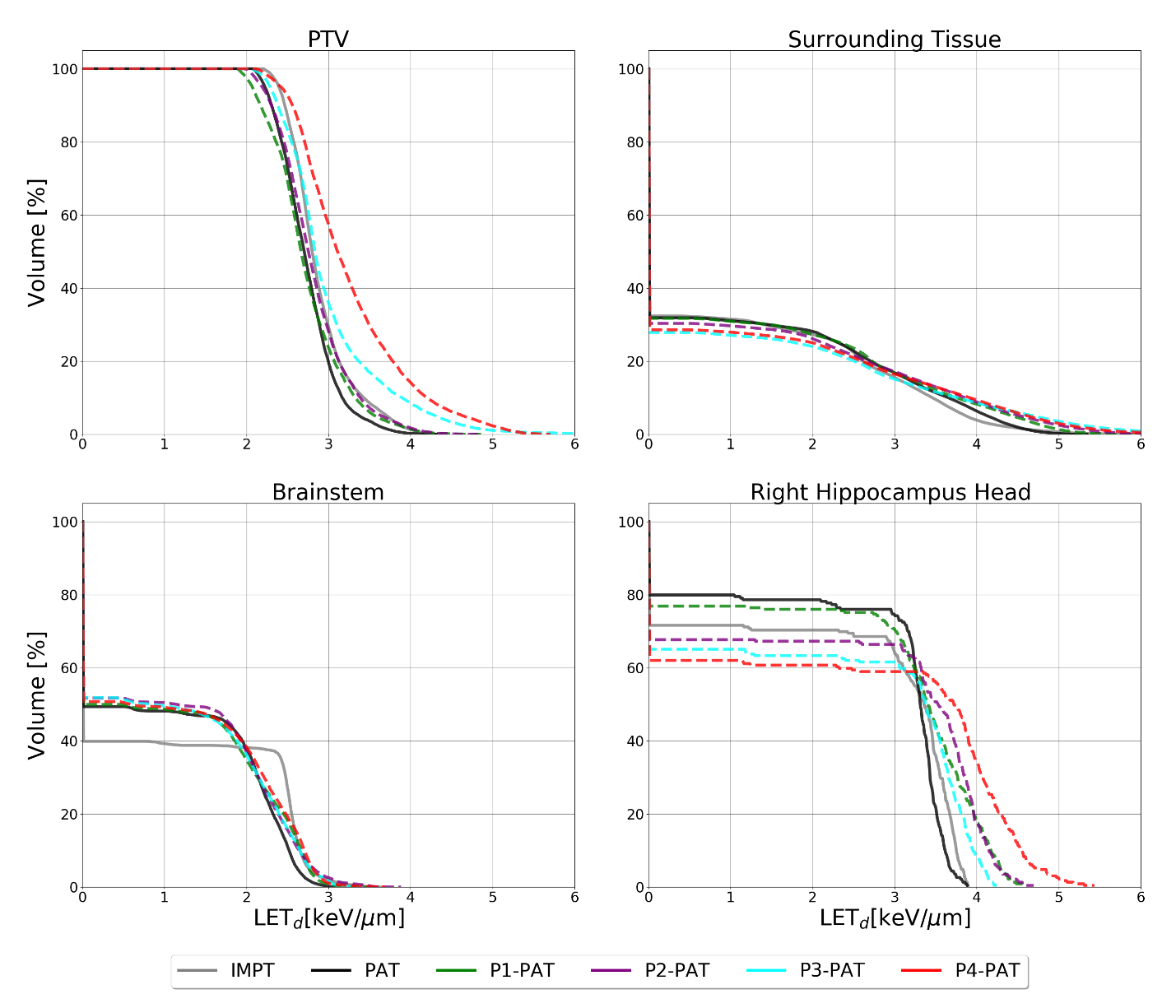


Figure A10 LET_d_ volume histogram for the PTV and OARs calculated with 30 Gy(RBE_1.1_) dose cutoffs for the ependymoma case. The dashed lines represent the P-PAT plans, while the solid lines represent the PAT plan.


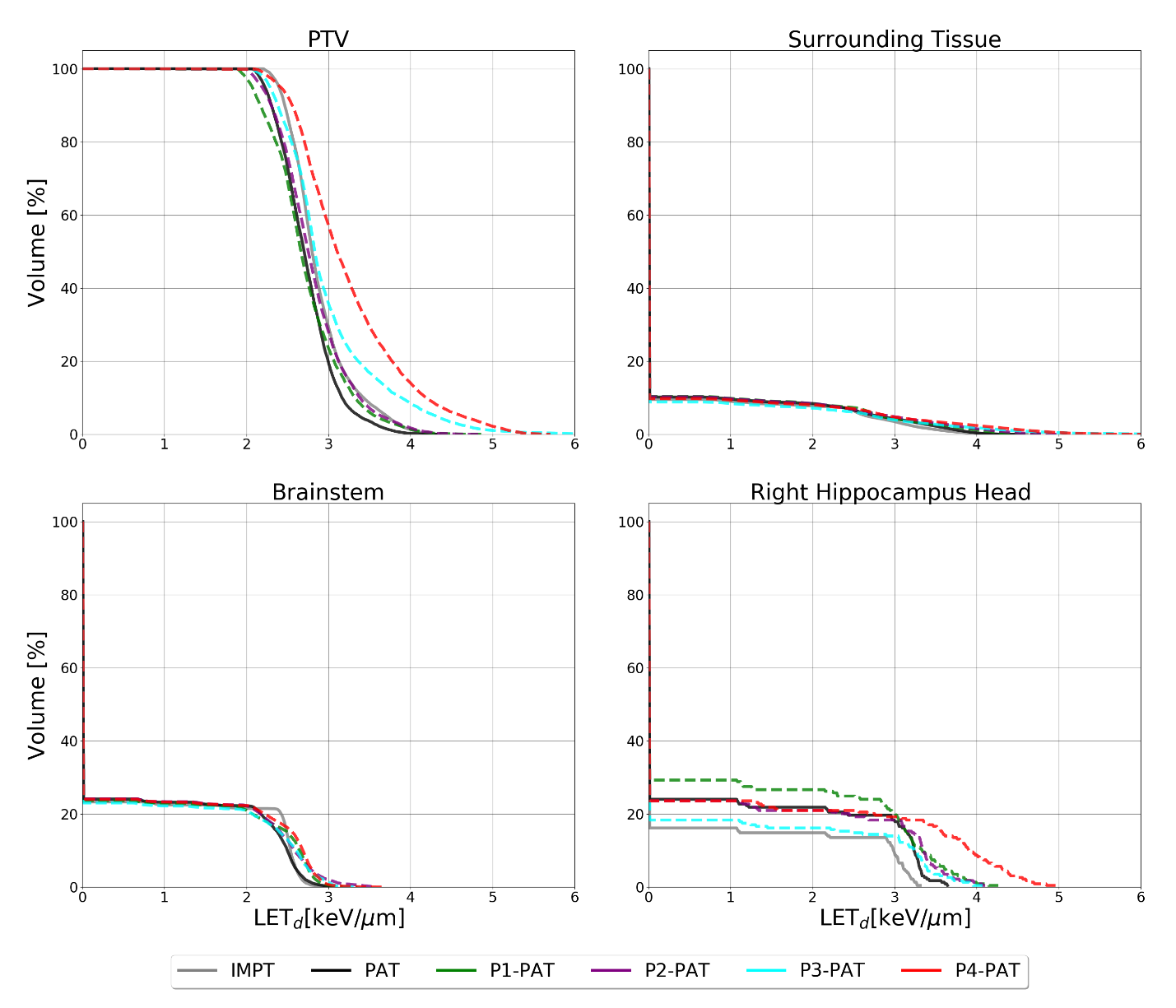


Figure A11 LET_d_ volume histogram for the PTV and OARs calculated with 50 Gy(RBE_1.1_) dose cutoffs for the ependymoma case. The dashed lines represent the P-PAT plans, while the solid lines represent the PAT plan.


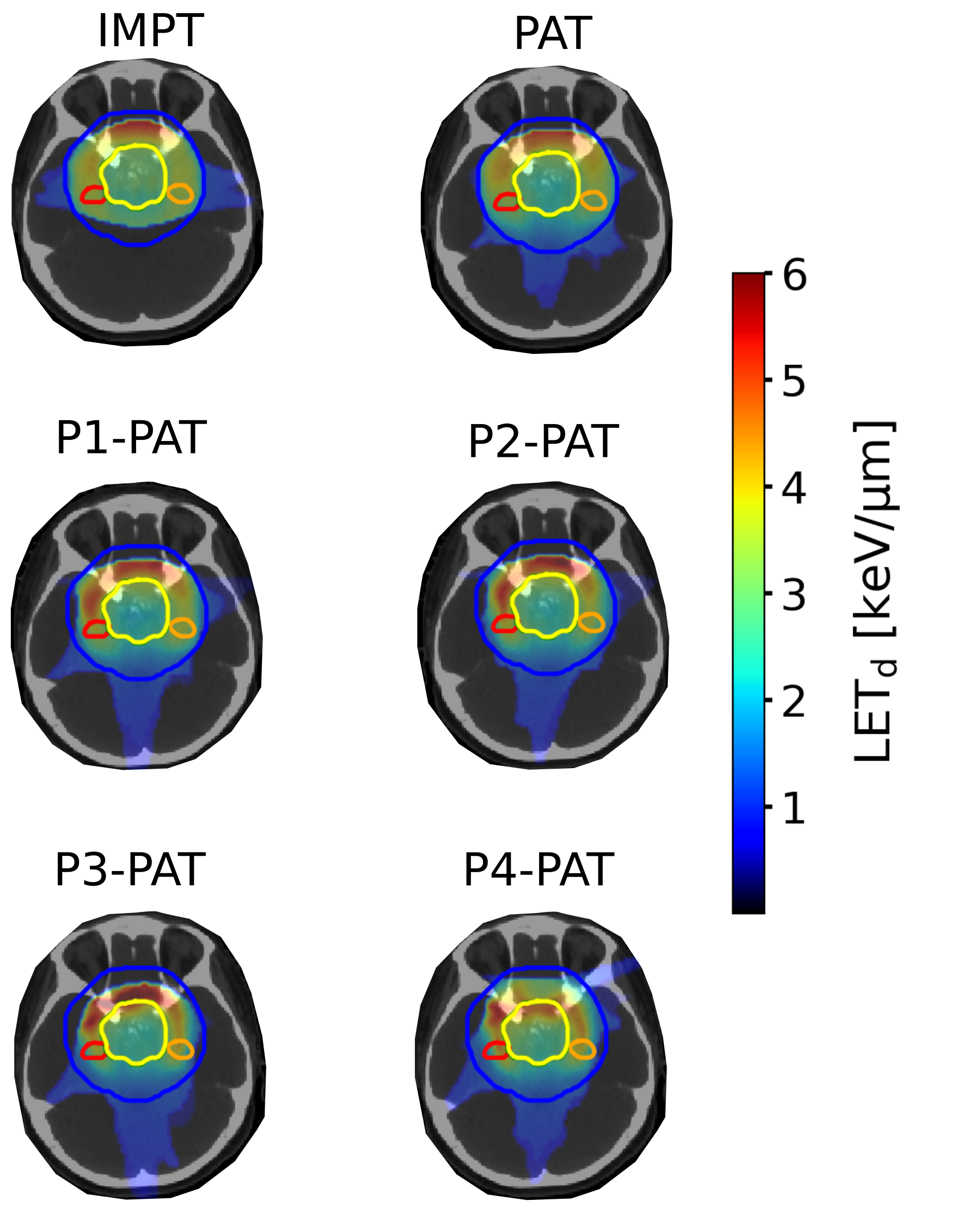


Figure A12 LET_d_ distribution for the different plans with an RBE_1.1_ weighted dose cutoff of 2 Gy(RBE) for the ependymoma case. The yellow contour represents the PTV, the surrounding blue contour represents the surrounding healthy tissue, and the red and orange represents the left and right hippocampi head, respectively.


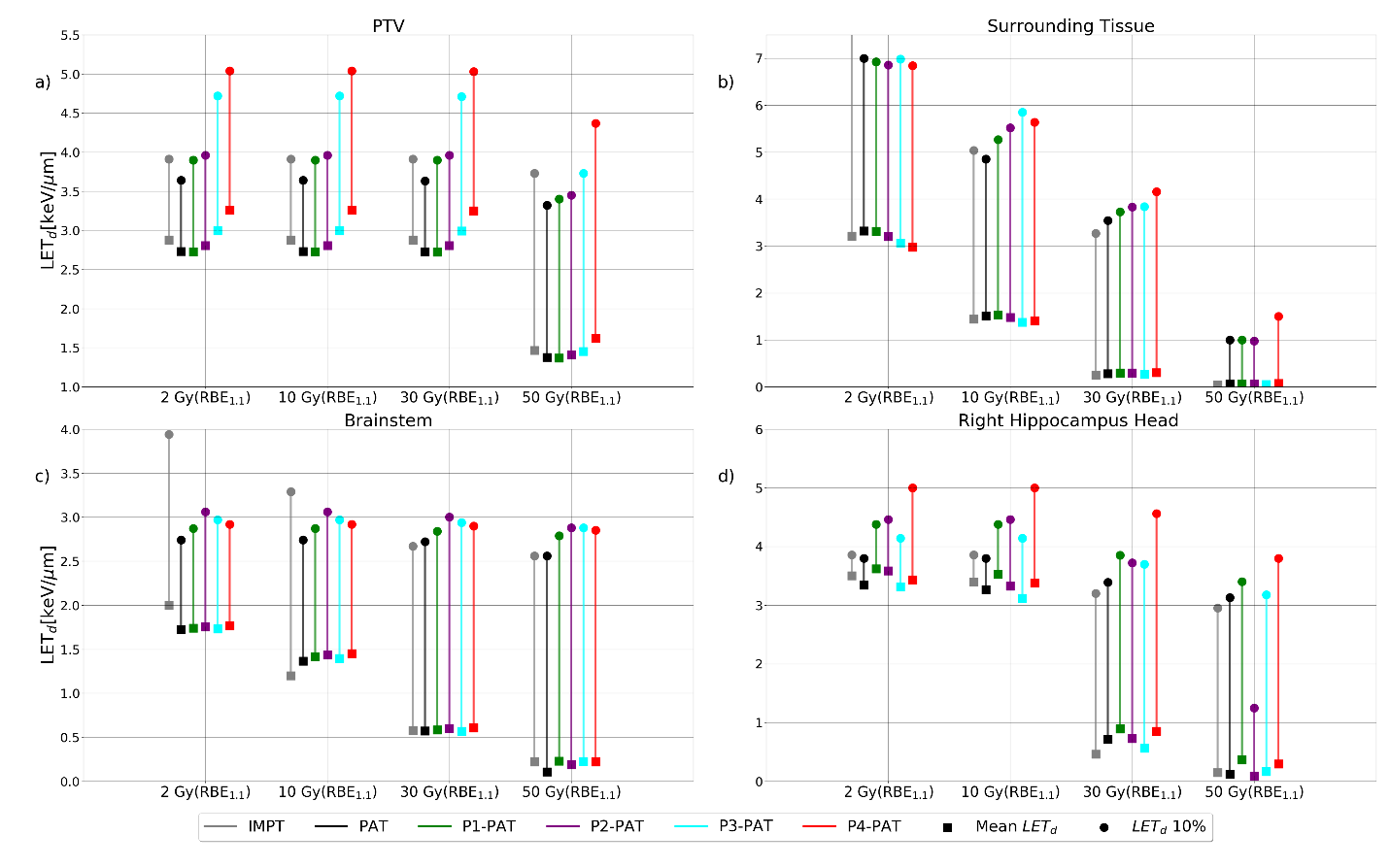


Figure A13 LET_d_ values for the PTV, surrounding healthy tissue and the OARs with different dose cutoff values for the ependymoma case. The square markers represent the mean LET_d_ and the circle markers represent the LET_d_ metrics for 2% of the volume.


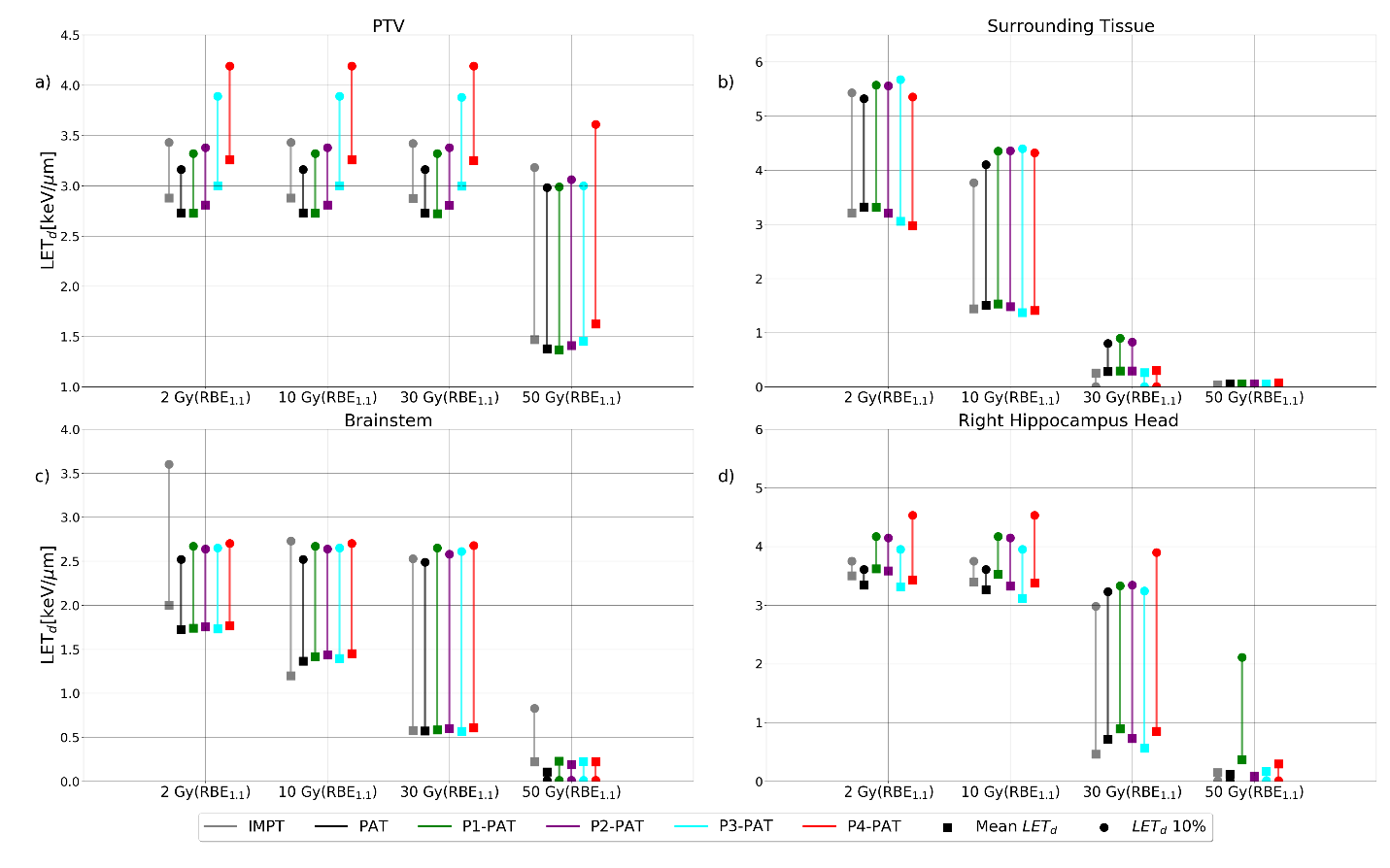


Figure A14 LET_d_ values for the PTV, surrounding healthy tissue and the OARs with different dose cutoff values for the ependymoma case. The square markers represent the mean LET_d_ and the circle markers represent the LET_d_ metrics for 10% of the volume.


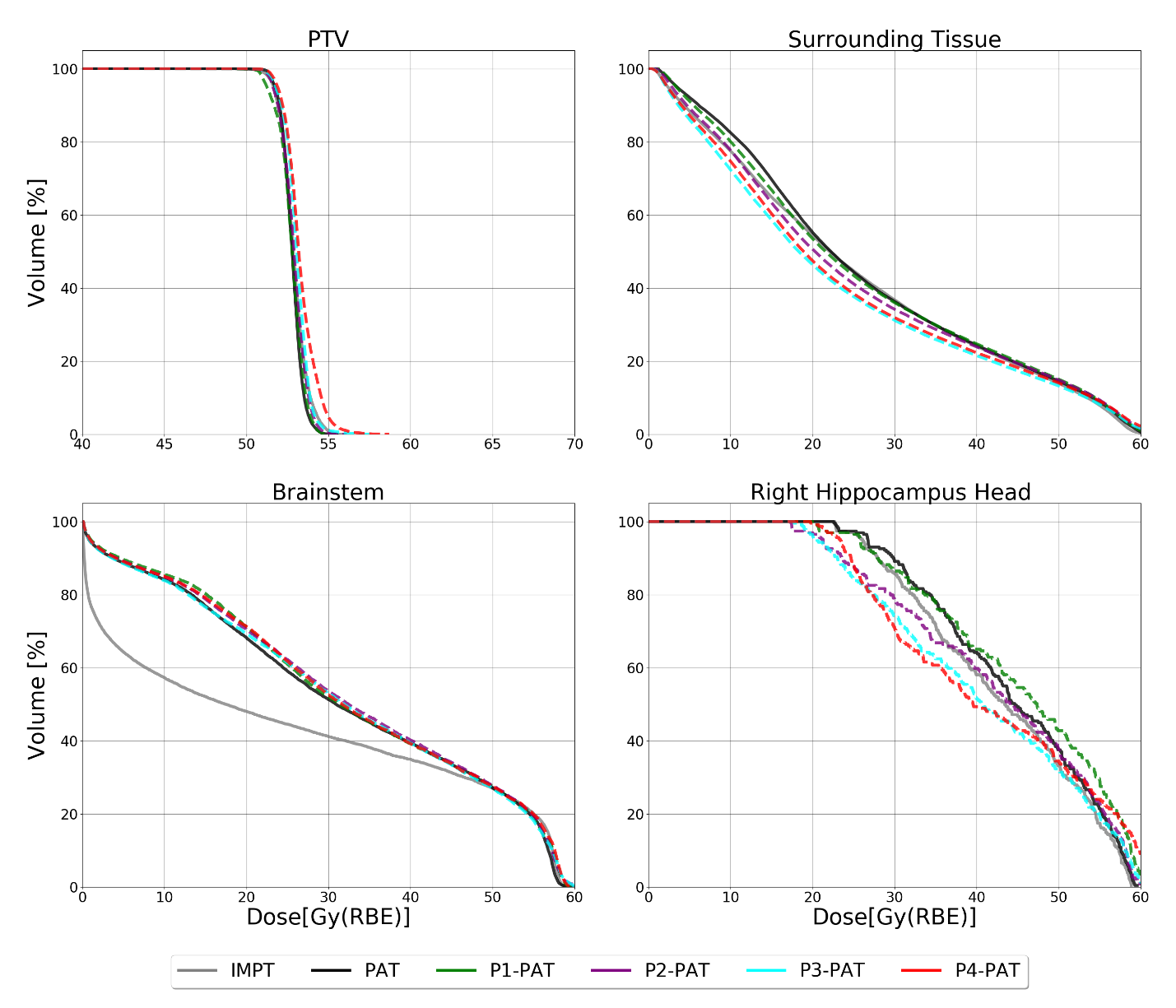


Figure A15 DVH for the ROR plan for the PTV, surrounding healthy tissue and the OARs for the ependymoma case. The dose from the PAT plan is represented by solid lines, while the P-PAT plans are represented with dashed lines.


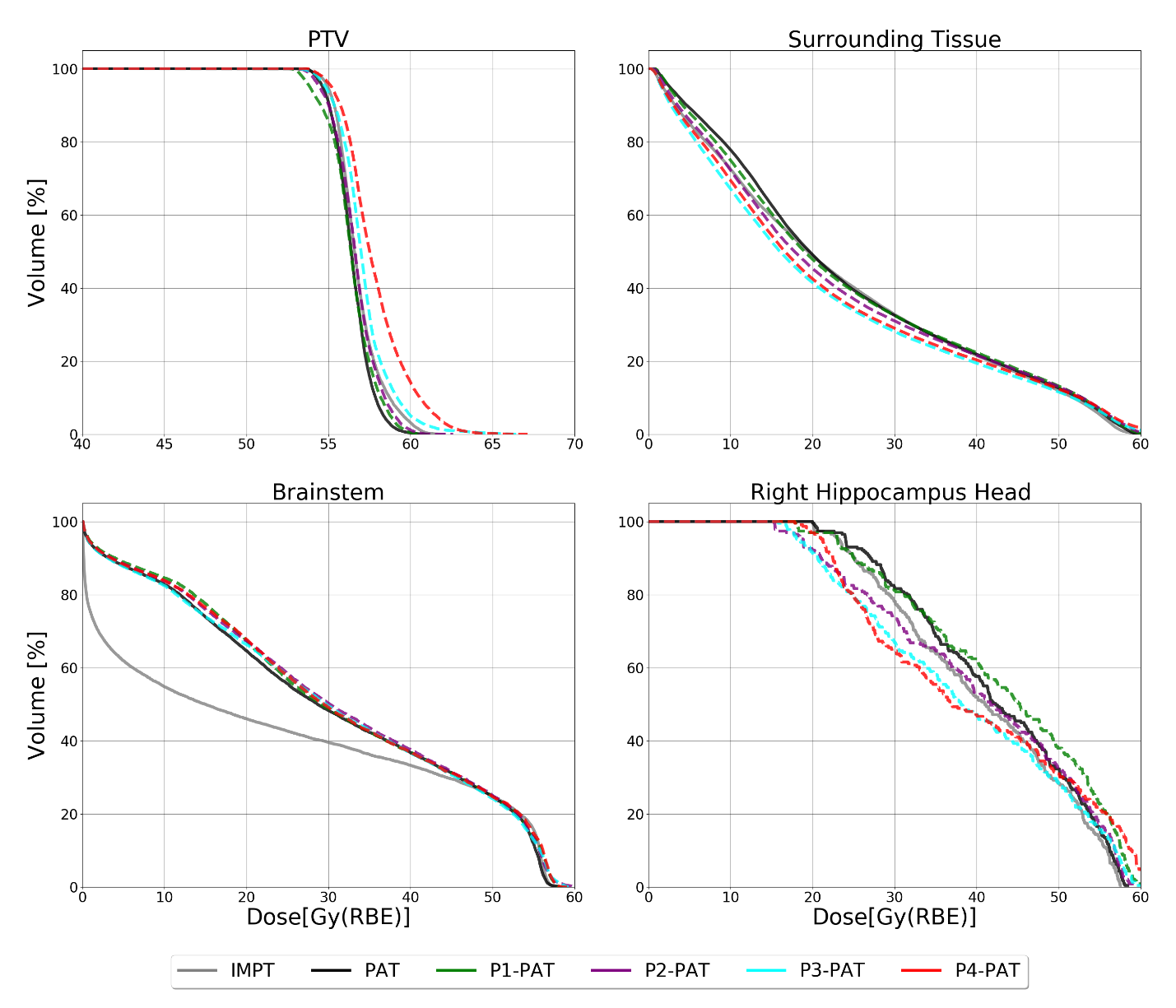


Figure A16 DVH for the LWD plan for the PTV, surrounding healthy tissue and the OARs for the ependymoma case. The dose from the PAT plan is represented by solid lines, while the P-PAT plans are represented with dashed lines.


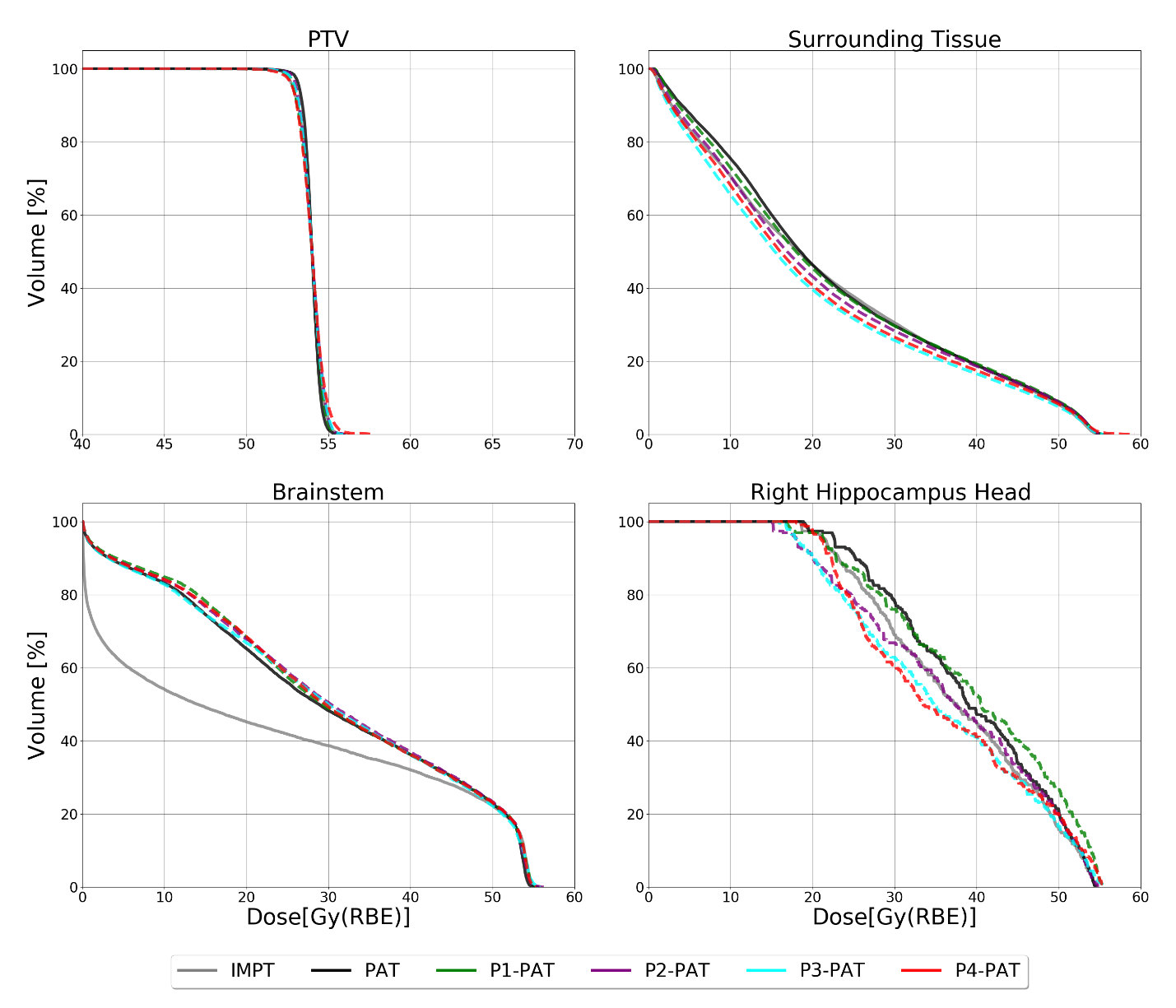


Figure A17 DVH for the RBE_1.1_ plan for the PTV, surrounding healthy tissue and the OARs for the ependymoma case. The dose from the PAT plan is represented by solid lines, while the P-PAT plans are represented with dashed lines.


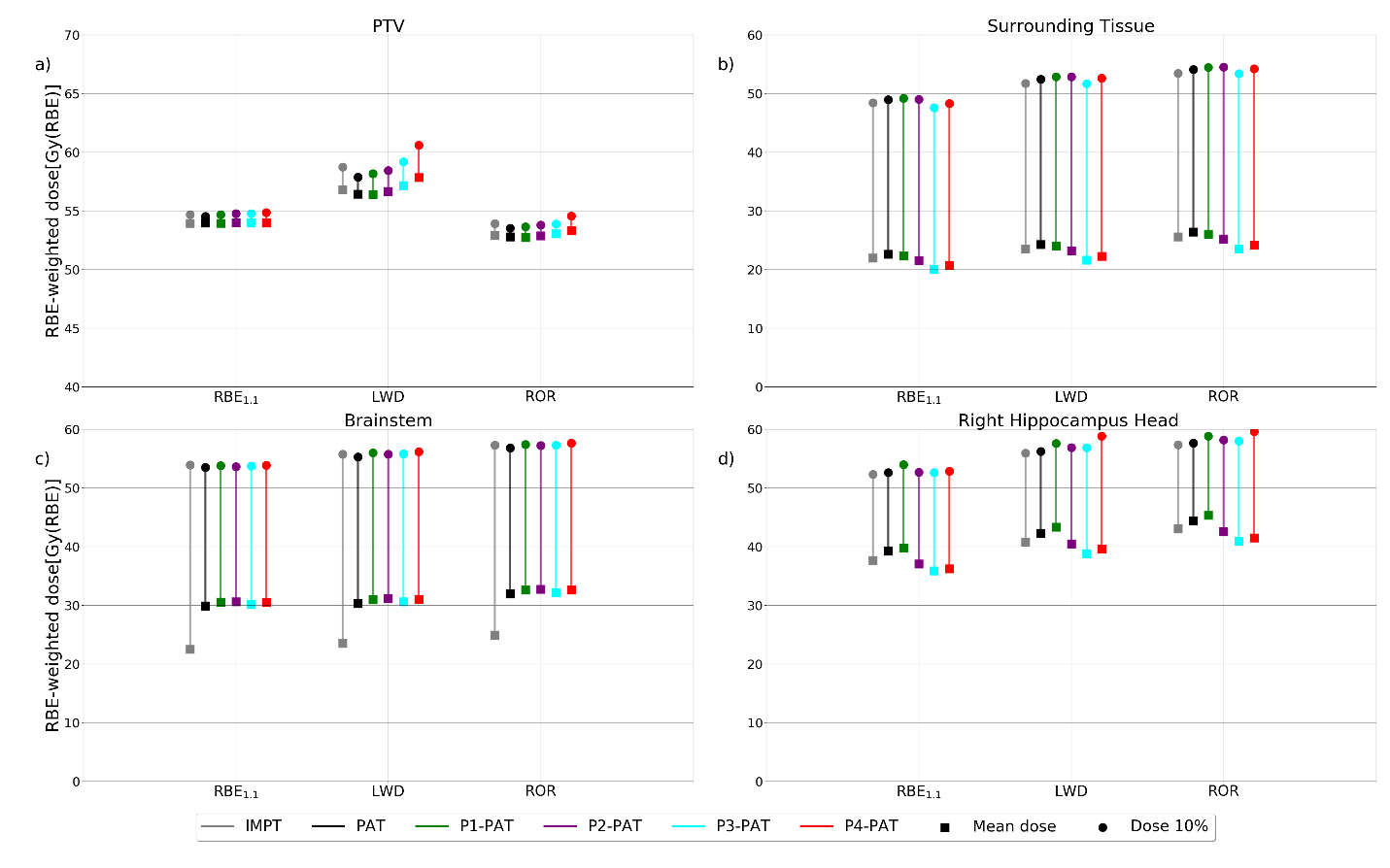


Figure A18 RBE-weighted dose values for the PTV, surrounding healthy tissue and the OARs for different RBE models for the ependymoma case. The square marker represents the mean RBE-weighted dose and the circle markers represent the RBE-weighted dose metrics for 2% of the volume.


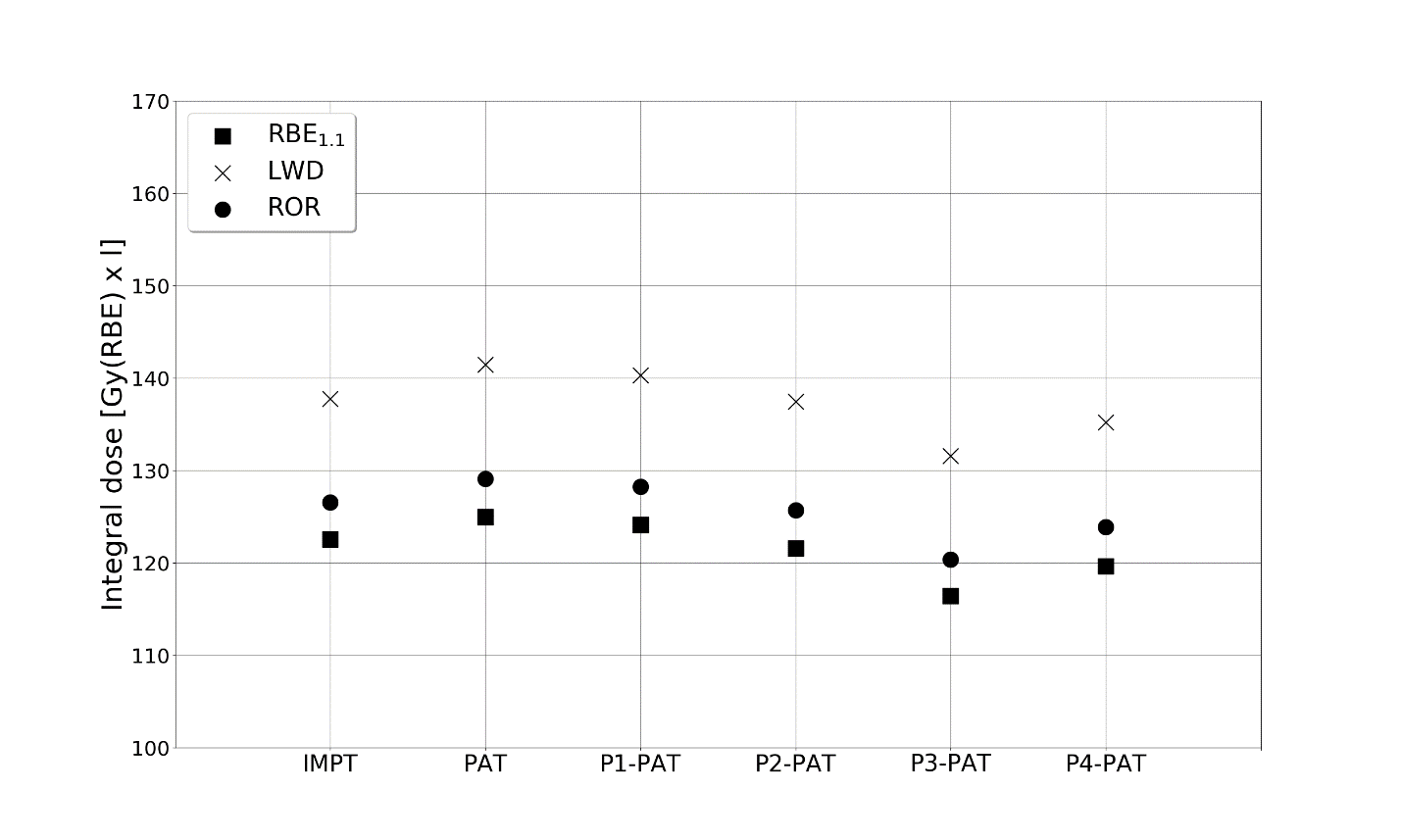


Figure A19 Integral doses for the different plans, where the different markers represent the different RBE-models for the ependymoma case. The integral doses are calculated as the total dose to a volume times the volume.

### **Additional results for the rhabdomyosarcoma case**

In Figure A20, Figure A21, and Figure A22, we see the LET_d_ volume histograms for the different dose cutoff-values, and in Figure A23, a colorwash of the LET_d_ for the different P-PAT plans are given. In Figure A24 and Figure A25 we see a scatterplot for the LET_d_ for different metrics. DVHs for the different RBE-models are given in Figure A26, Figure A27 and Figure A28, while Figure A29 show an additional scatterplot for the RBE weighted dose metrics. Figure A30 shows the integral dose for all RBE-models.


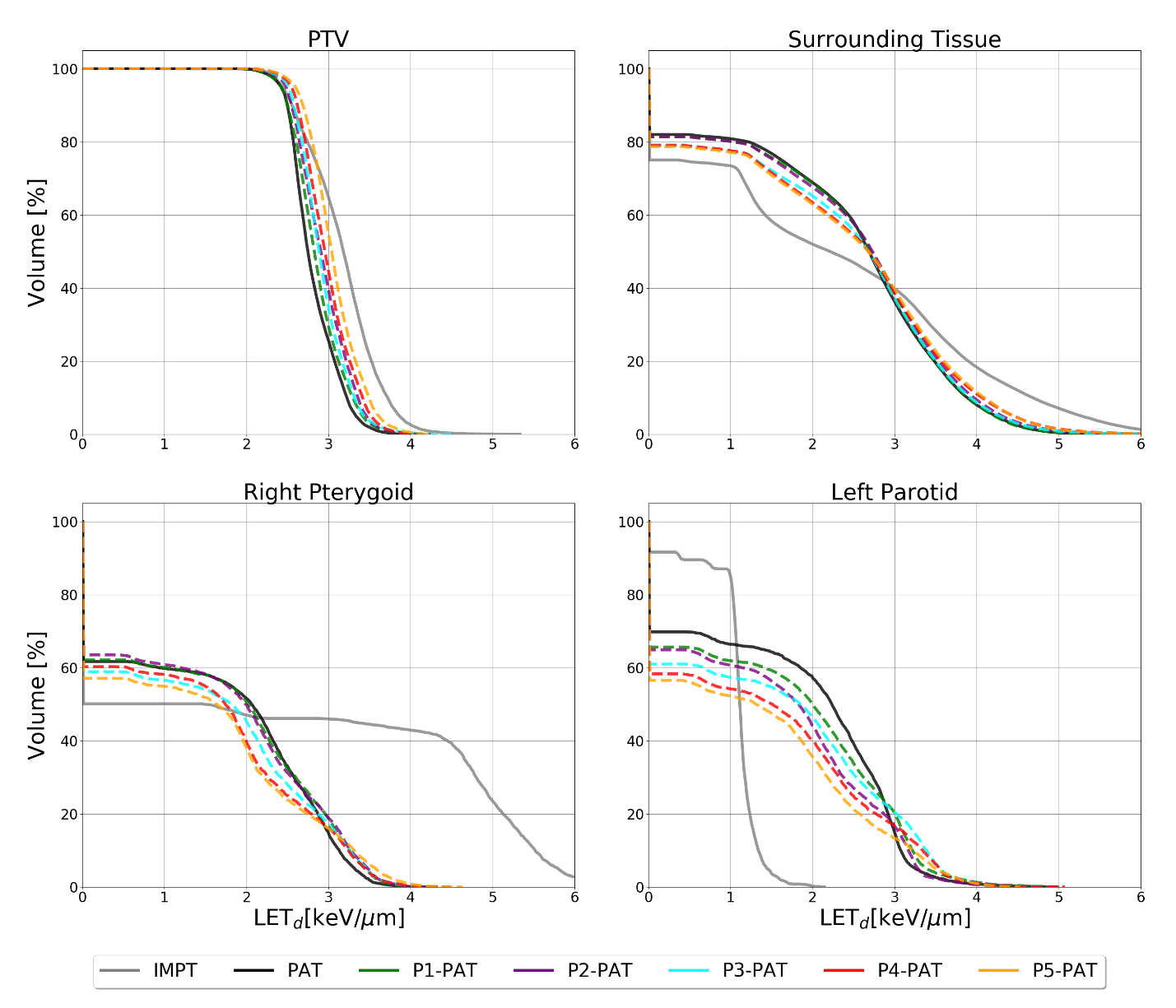


Figure A20 LET_d_ volume histogram for the PTV and OARs calculated with 10 Gy(RBE_1.1_) dose cutoffs for the rhabdomyosarcoma case. The dashed lines represent the P-PAT plans, while the solid lines represent the PAT plan.


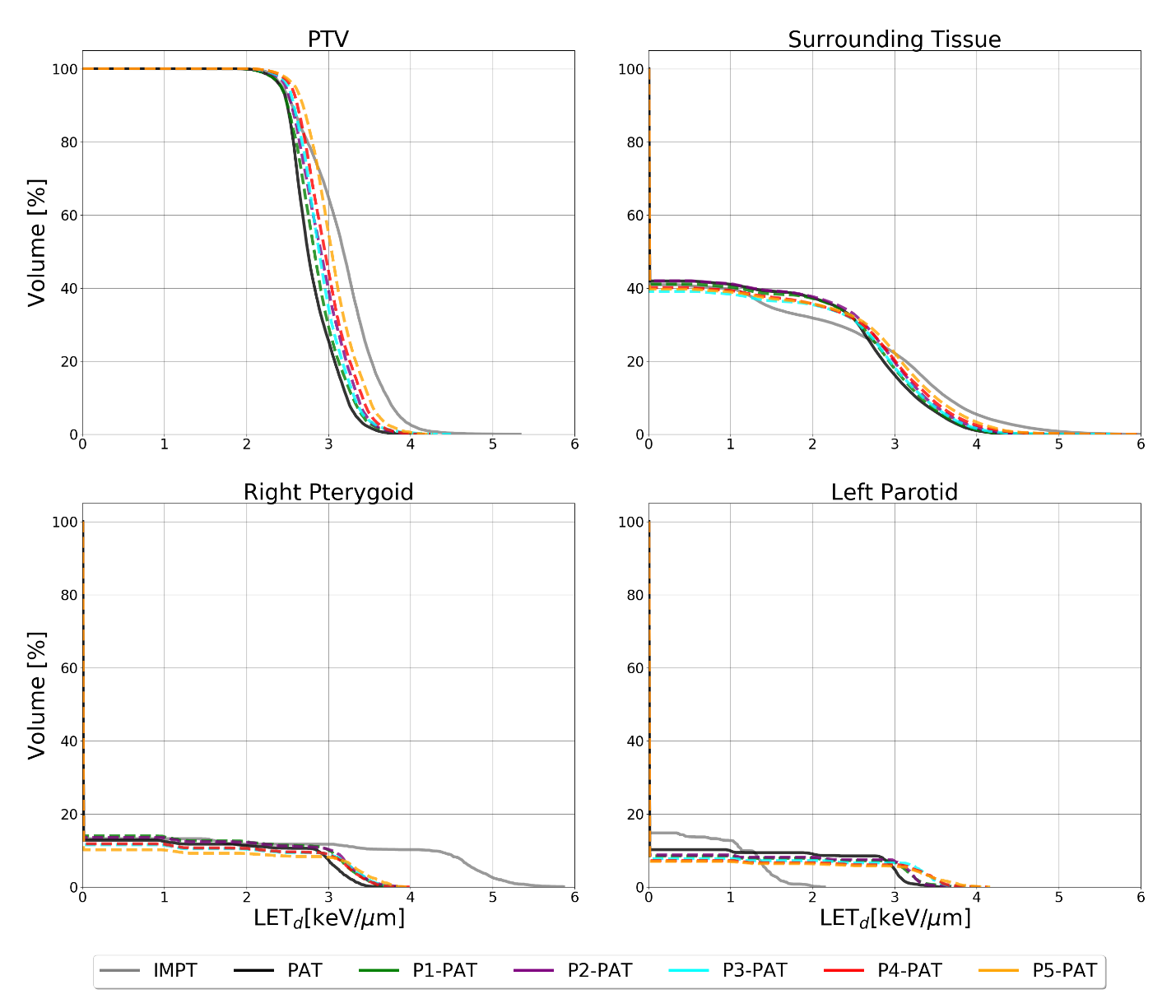


Figure A21 LET_d_ volume histogram for the PTV and OARs calculated with 10 Gy(RBE_1.1_) dose cutoffs for the rhabdomyosarcoma case. The dashed lines represent the P-PAT plans, while the solid lines represent the PAT plan.


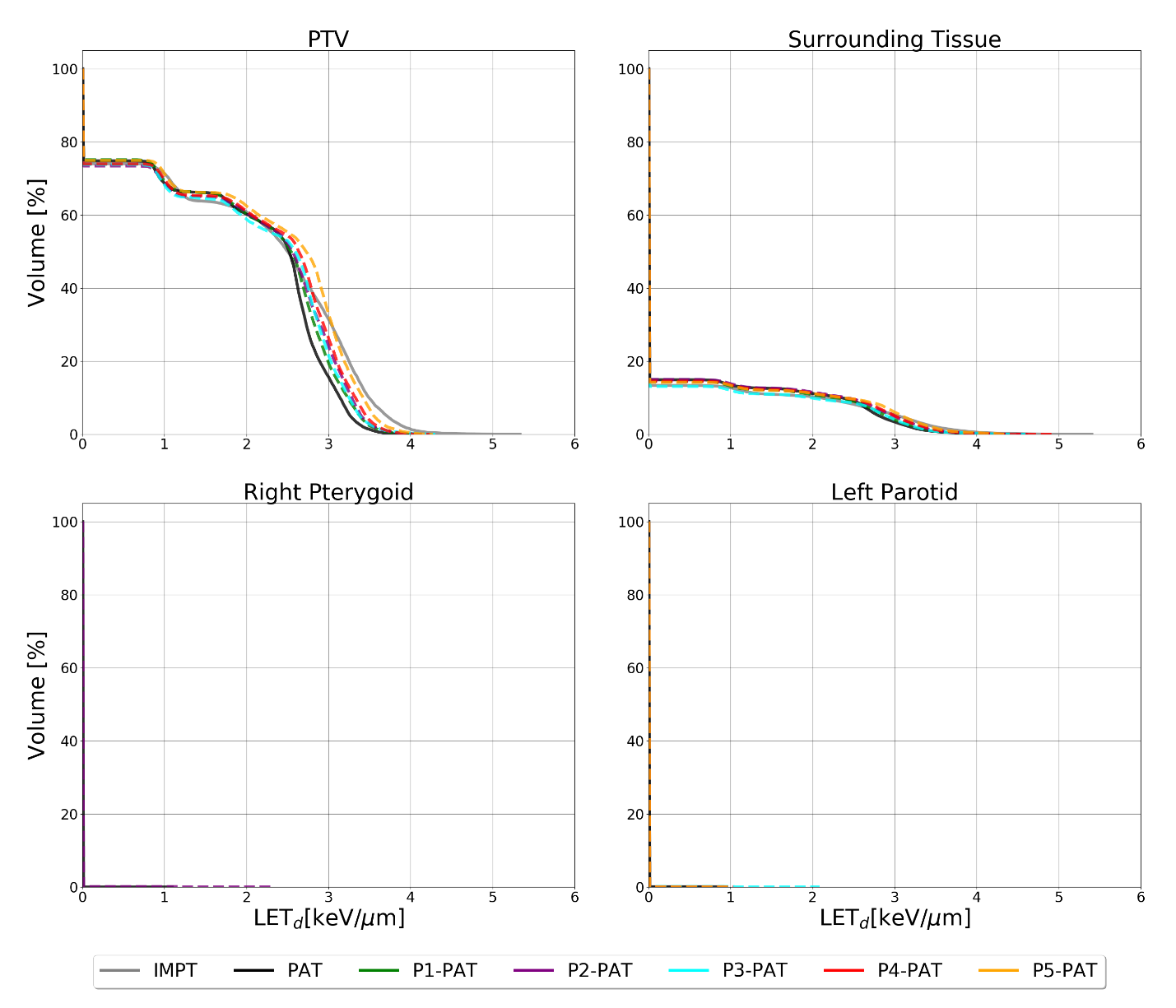


Figure A22 LET_d_ volume histogram for the PTV and OARs calculated with 10 Gy(RBE_1.1_) dose cutoffs for the rhabdomyosarcoma case. The dashed lines represent the P-PAT plans, while the solid lines represent the PAT plan.


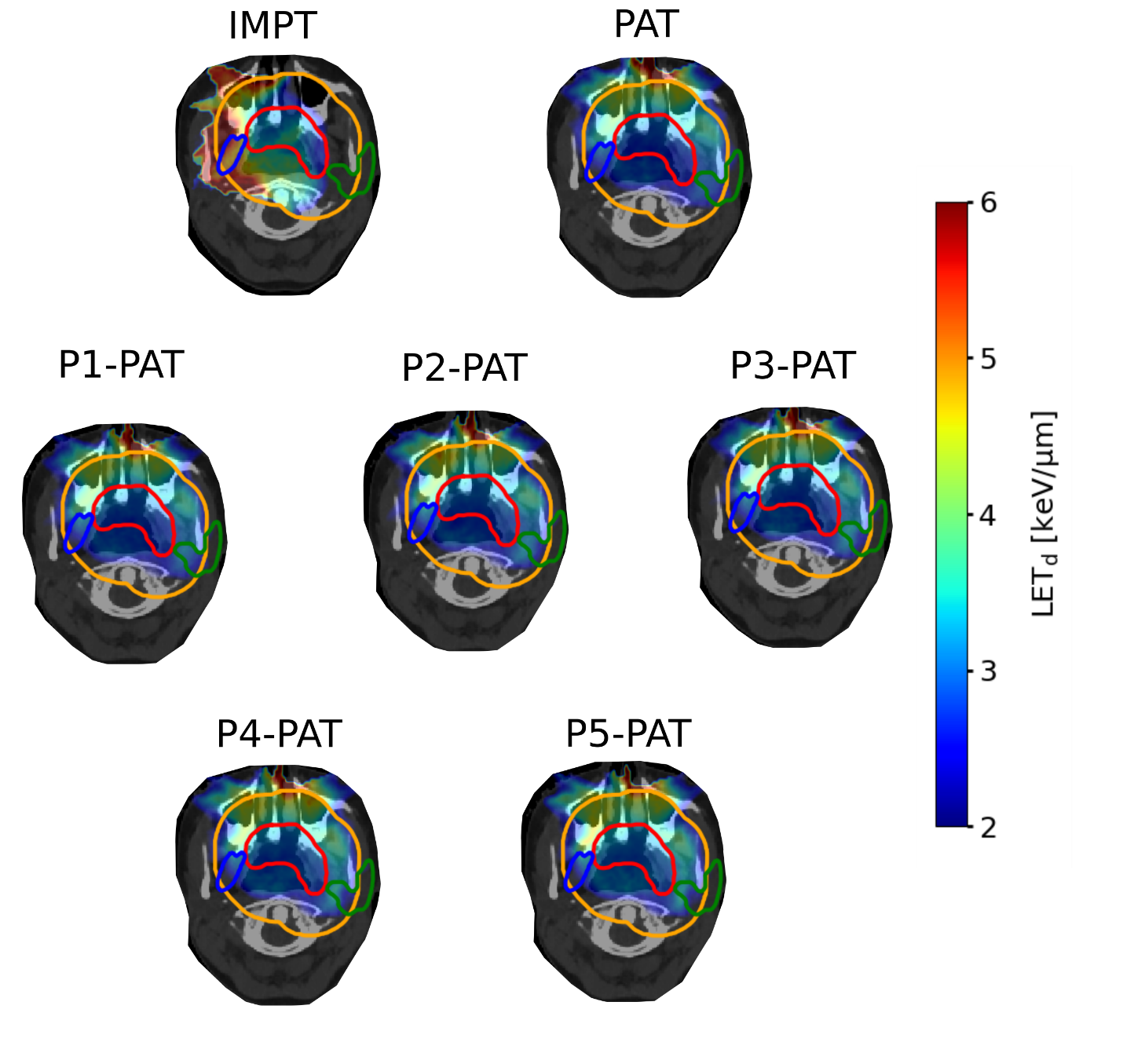


Figure A23 LET_d_ distribution for the different plans with an RBE_1.1_ weighted dose cutoff of 2 Gy(RBE) for the rhabdomyosarcoma case. The red contour represents the PTV, the surrounding orange contour represents the surrounding healthy tissue, the bluel represents the right pterygoid and the green represent the left parotid.


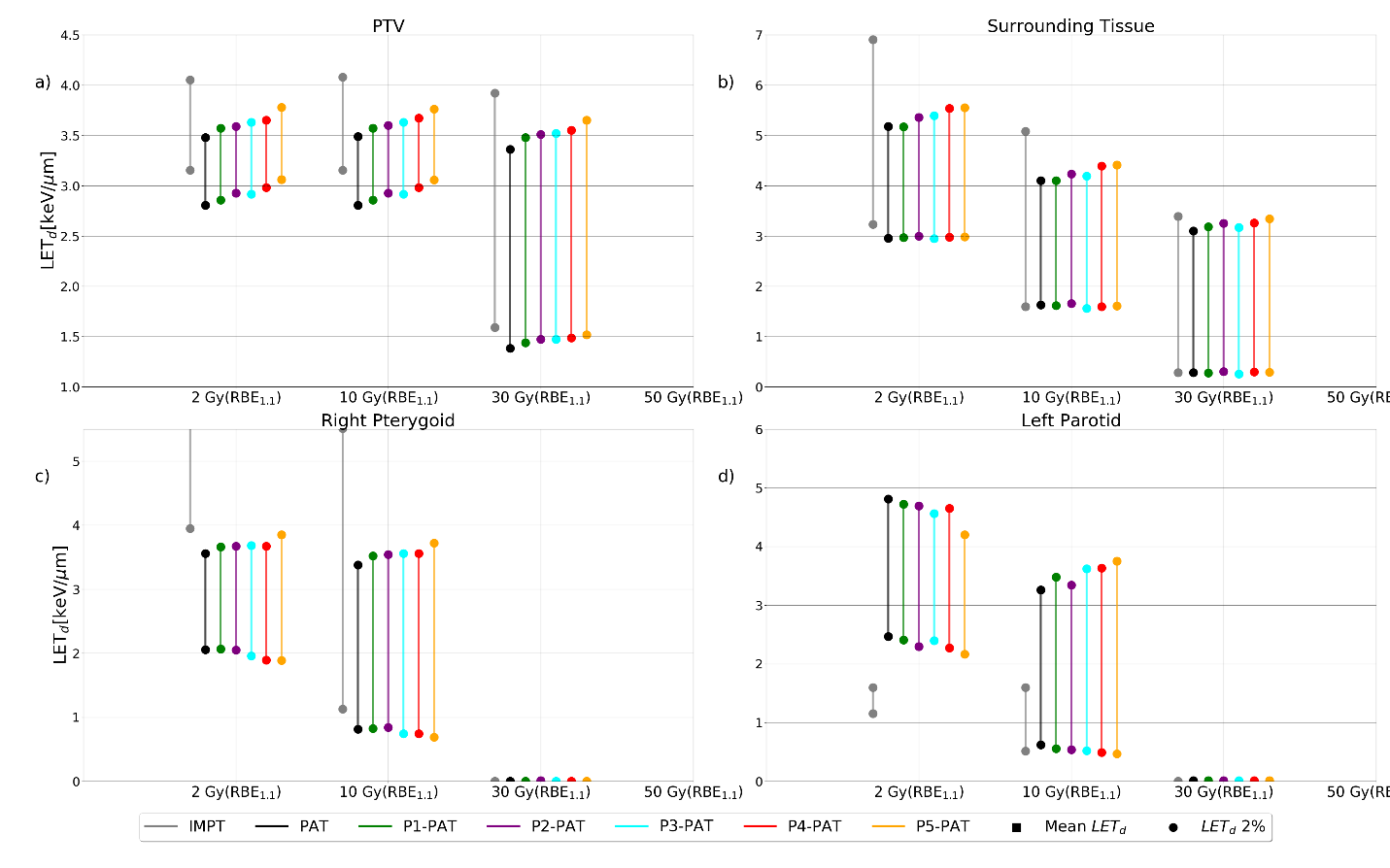


Figure A24 LET_d_ values for the PTV, surrounding healthy tissue and the OARs with different dose cutoff values for the rhabdomyosarcoma case. The square markers represent the mean LET_d_ and the circle markers represent the LET_d_ metrics for 2% of the volume.


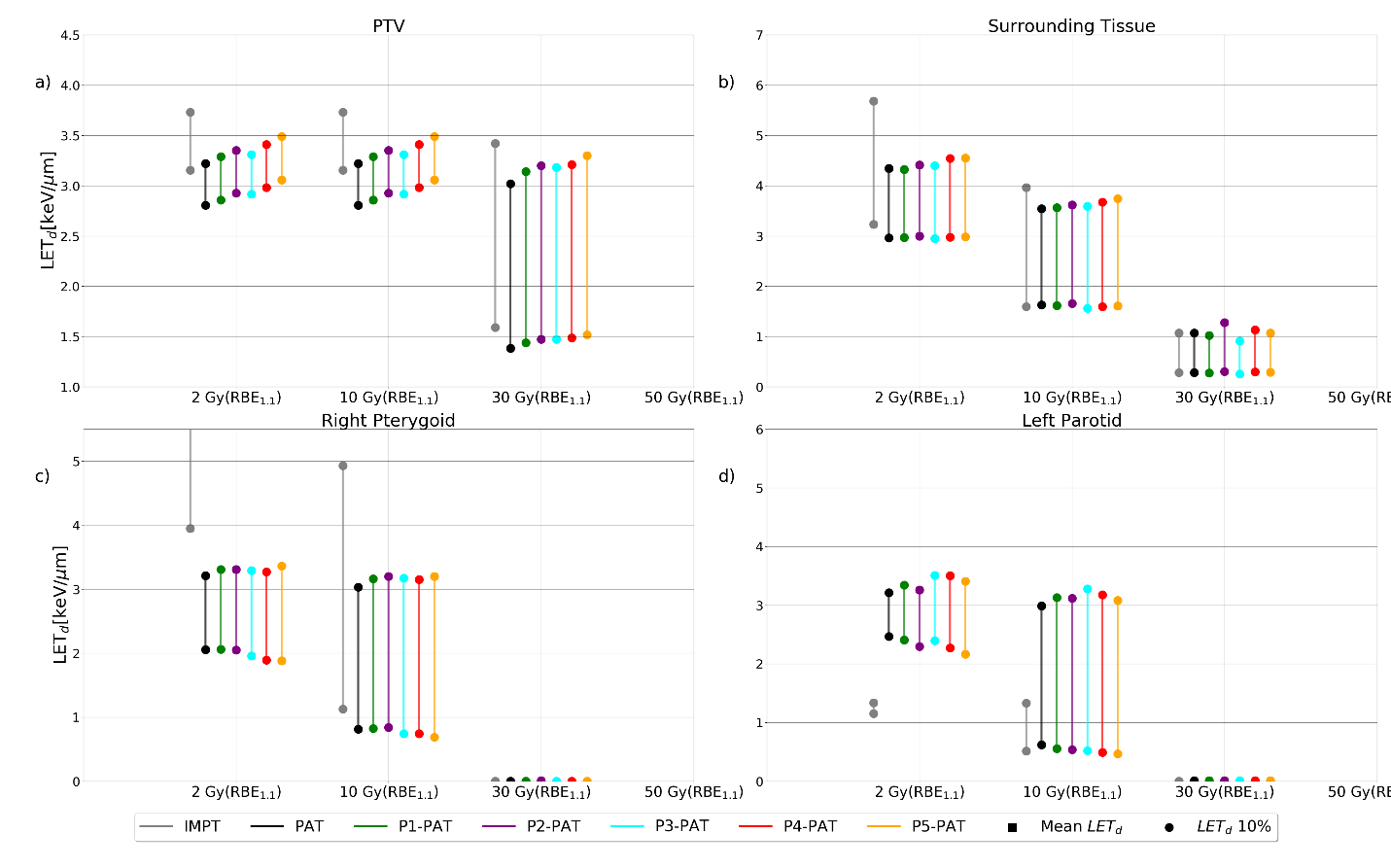


Figure A25 LET_d_ values for the PTV, surrounding healthy tissue and the OARs with different dose cutoff values for the rhabdomyosarcoma case. The square markers represent the mean LET_d_ and the circle markers represent the LET_d_ metrics for 10% of the volume.


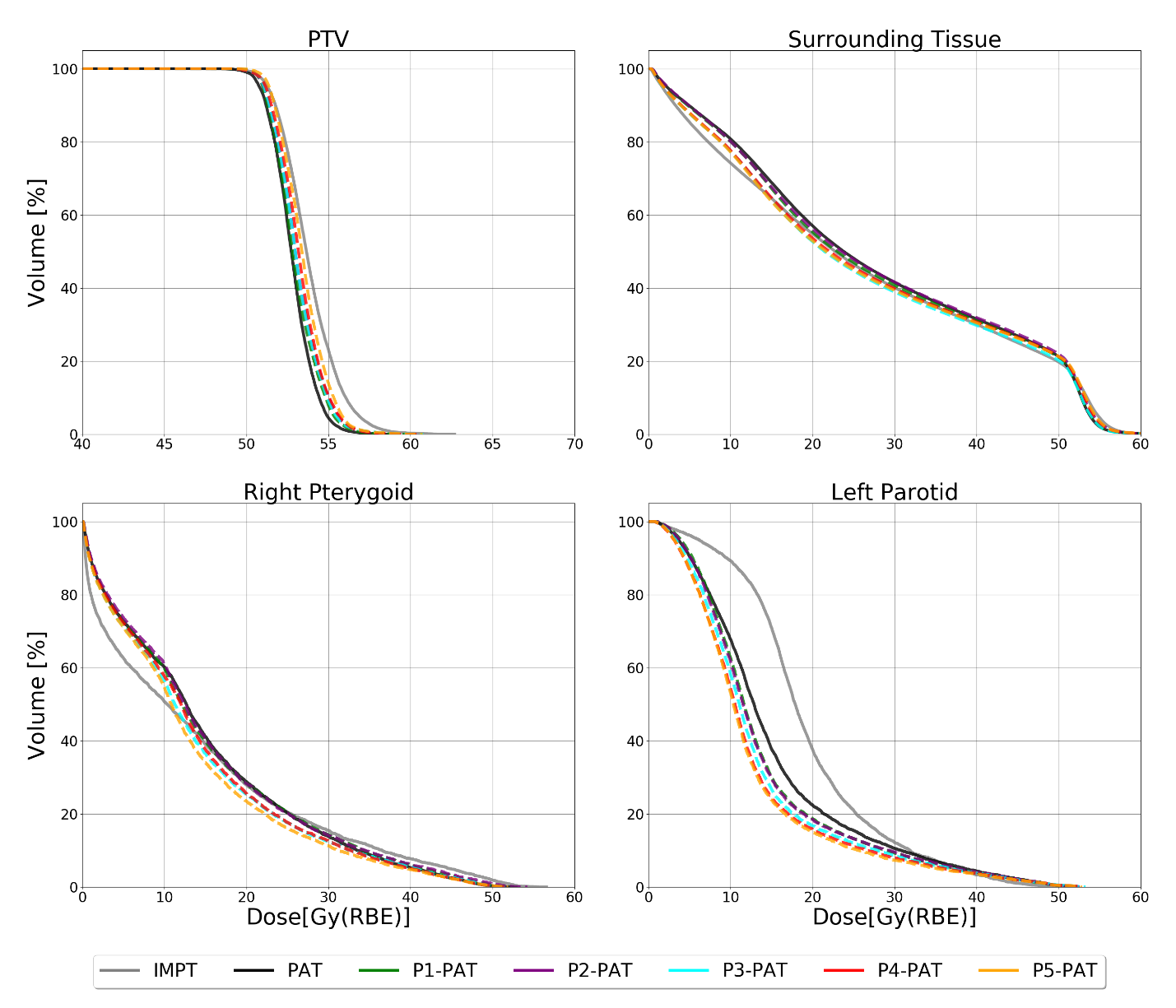


Figure A26 DVH for the ROR plan for the PTV, surrounding healthy tissue and the OARs for the rhabdomyosarcoma case. The dose from the PAT plan is represented by solid lines, while the P-PAT plans are represented with dashed lines.


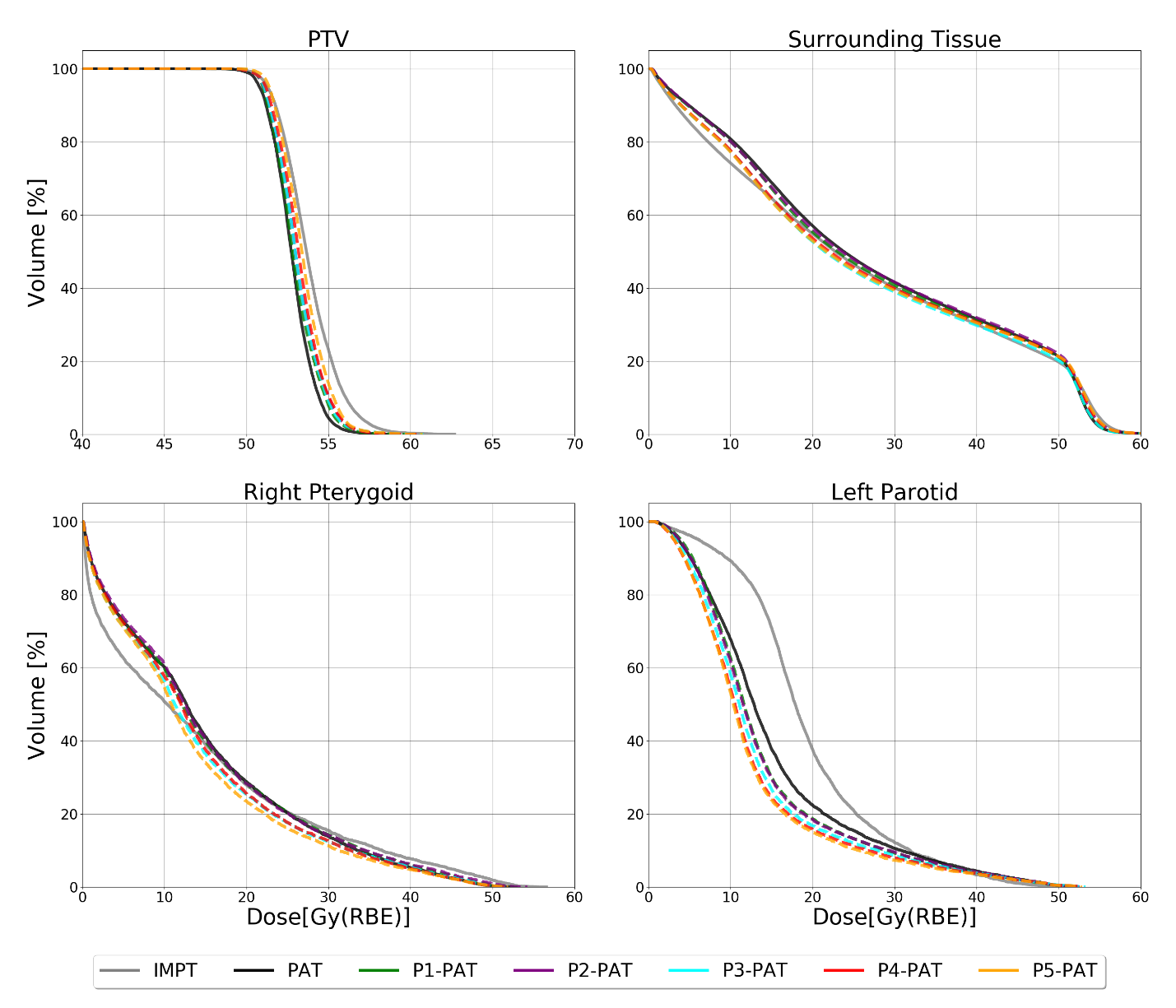


Figure A27 DVH for the LWD plan for the PTV, surrounding healthy tissue and the OARs for the rhabdomyosarcoma case. The dose from the PAT plan is represented by solid lines, while the P-PAT plans are represented with dashed lines.


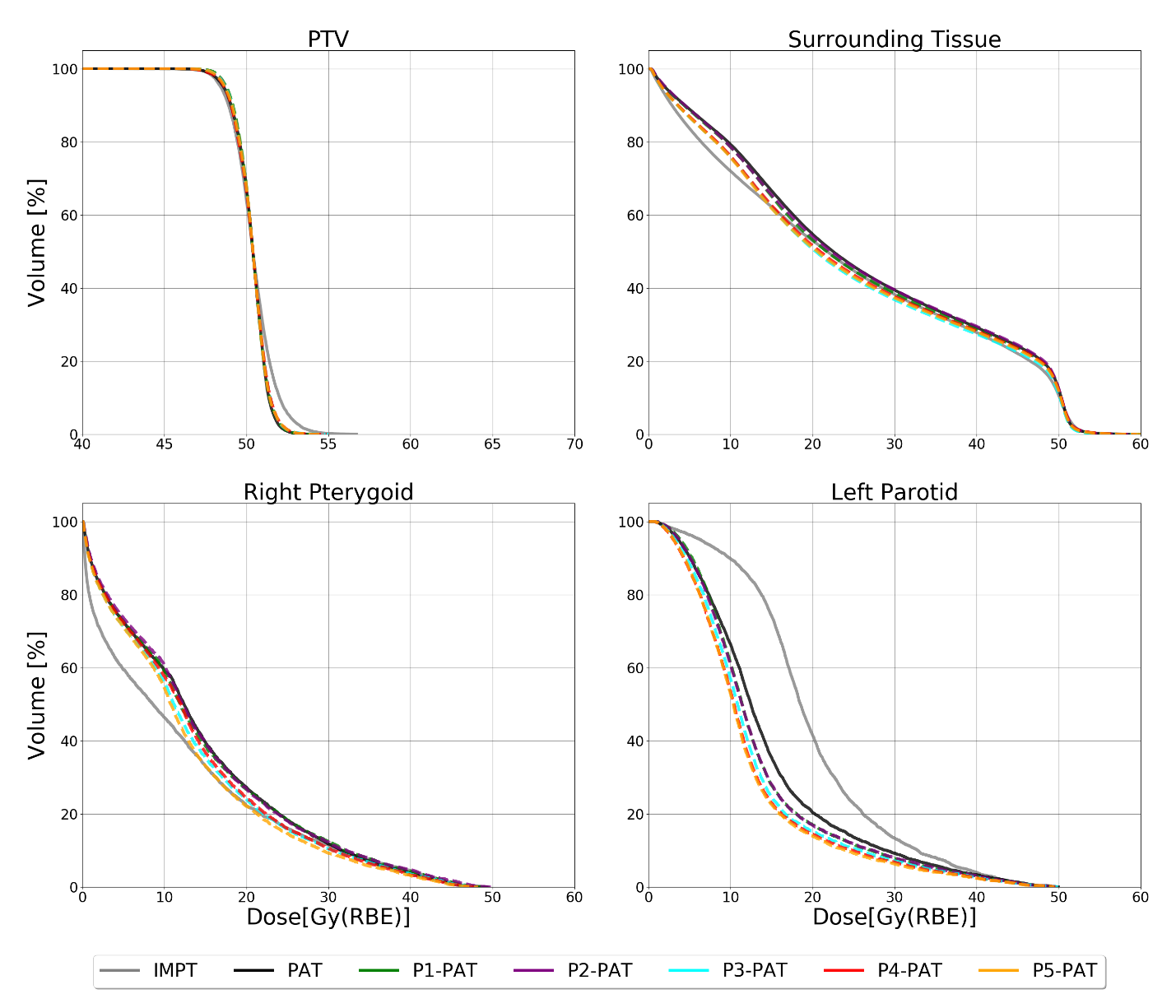


Figure A28 DVH for the RBE_1.1_ plan for the PTV, surrounding healthy tissue and the OARs for the rhabdomyosarcoma case. The dose from the PAT plan is represented by solid lines, while the P-PAT plans are represented with dashed lines.


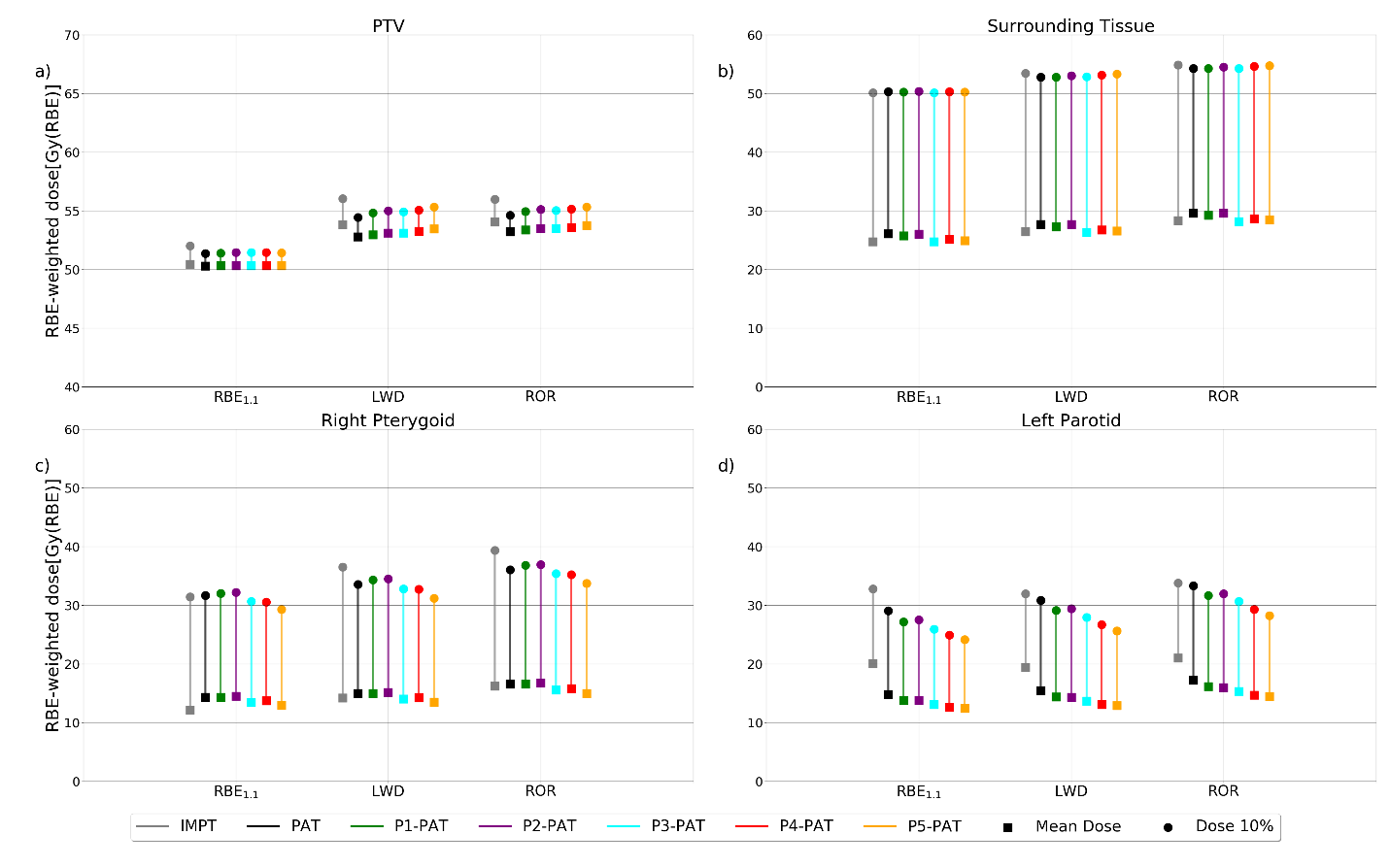


Figure A29 RBE-weighted dose values for the PTV, surrounding healthy tissue and the OARs for different RBE models for the rhabdomyosarcoma case. The square marker represents the mean RBE-weighted dose and the circle markers represent the RBE-weighted dose metrics for 10% of the volume.


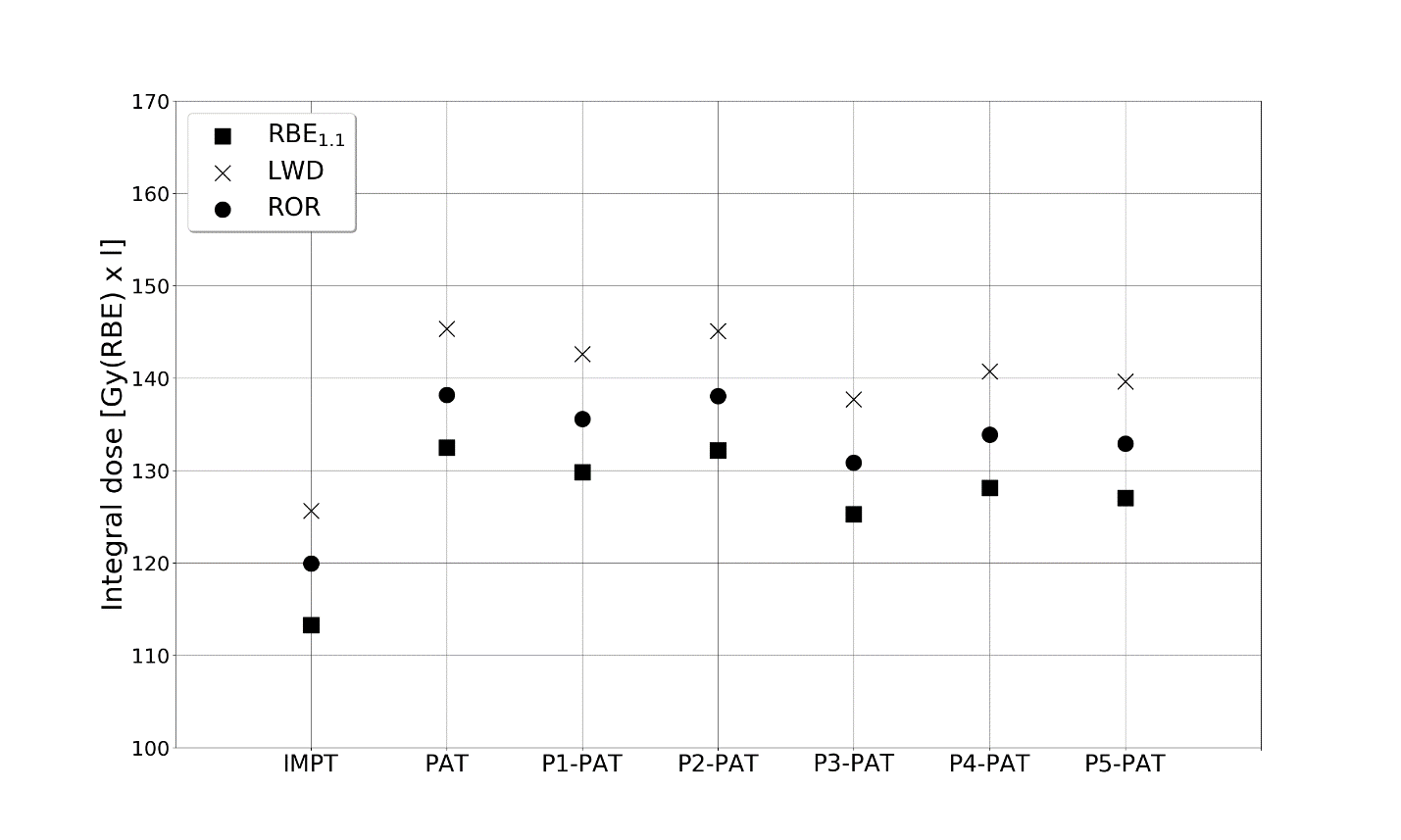


Figure A30 Integral doses for the different plans, where the different markers represent the different RBE-models for the rhabdomyosarcoma case. The integral doses are calculated as the total dose to a volume times the volume.
